# Patterns and processes of pathogen exposure in gray wolves across North America

**DOI:** 10.1038/s41598-021-81192-w

**Published:** 2021-02-12

**Authors:** Ellen E. Brandell, Paul C. Cross, Meggan E. Craft, Douglas W. Smith, Edward J. Dubovi, Marie L. J. Gilbertson, Tyler Wheeldon, John A. Stephenson, Shannon Barber-Meyer, Bridget L. Borg, Mathew Sorum, Daniel R. Stahler, Allicia Kelly, Morgan Anderson, H. Dean Cluff, Daniel R. MacNulty, Dominique E. Watts, Gretchen H. Roffler, Helen Schwantje, Mark Hebblewhite, Kimberlee Beckmen, Heather Fenton, Peter J. Hudson

**Affiliations:** 1 Center for Infectious Disease Dynamics, Department of Biology, Huck Institutes of the Life Sciences, Pennsylvania State University, University Park, PA 16802 USA; 2 U.S. Geological Survey, Northern Rocky Mountain Science Center, Bozeman, MT 59715 USA; 3 Department of Ecology, Evolution, and Behavior, University of Minnesota, Saint Paul, MN 55108 USA; 4 Yellowstone Center for Resources, Wolf Project, P.O. Box 168, Yellowstone National Park, WY 82190 USA; 5 Animal Health Diagnostic Center, College of Veterinary Medicine, Cornell University, Ithaca, NY 14850 USA; 6 Department of Veterinary Population Medicine, University of Minnesota, Saint Paul, MN 55108 USA; 7 Ontario Ministry of Natural Resources and Forestry, Trent University, 2140 East Bank Drive, Peterborough, ON K9L 1Z8 Canada; 8 Grand Teton National Park, P.O. Drawer 170, Moose, WY 83012 USA; 9 U.S. Geological Survey, Northern Prairie Wildlife Research Center, 8711 37th St. SE, Jamestown, ND 58401 USA; 10 Denali National Park and Preserve, Central Alaska Inventory and Monitoring Network, P.O. Box 9, Denali Park, AK 99755 USA; 11 Yukon-Charley Rivers National Preserve, Central Alaska Inventory and Monitoring Network, 4175 Geist Road, Fairbanks, AK 99709 USA; 12 Department of Environment and Natural Resources, Government of the Northwest Territories, P.O. Box 900, Fort Smith, NT X0E 0P0 Canada; 13 British Columbia Ministry of Forests, Lands, Natural Resource Operations and Rural Development, 2000 South Ospika Blvd., Prince George, BC V2N 4W5 Canada; 14 Environment and Natural Resources, Government of the Northwest Territories, North Slave Region, NT X1A 2P9 Canada; 15 Department of Wildland Resources, Utah State University, Logan, UT 84322 USA; 16 U.S. Fish and Wildlife Service, Kenai National Wildlife Refuge, P.O. 2139, Soldotna, AK 99669 USA; 17 Division of Wildlife Conservation, Alaska Department of Fish and Game, 802 3rd Street, Douglas, AK 99824 USA; 18 Wildlife and Habitat Branch, British Columbia Ministry of Forests, Lands, Natural Resource Operations and Rural Development, 2080 Labieux Road, Nanaimo, BC V9T 6J9 Canada; 19 Wildlife Biology Program, Department of Ecosystem and Conservation Sciences, Franke College of Forestry and Conservation, University of Montana, Missoula, MT 59812 USA; 20 Division of Wildlife Conservation, Dept of Fish and Game, 1300 College Road, Fairbanks, AK 99701 USA; 21 Ross University School of Veterinary Medicine, Basseterre, West Indies KN-03 St. Kitts and Nevis

**Keywords:** Ecology, Ecological epidemiology

## Abstract

The presence of many pathogens varies in a predictable manner with latitude, with infections decreasing from the equator towards the poles. We investigated the geographic trends of pathogens infecting a widely distributed carnivore: the gray wolf (*Canis lupus*). Specifically, we investigated which variables best explain and predict geographic trends in seroprevalence across North American wolf populations and the implications of the underlying mechanisms. We compiled a large serological dataset of nearly 2000 wolves from 17 study areas, spanning 80° longitude and 50° latitude. Generalized linear mixed models were constructed to predict the probability of seropositivity of four important pathogens: canine adenovirus, herpesvirus, parvovirus, and distemper virus—and two parasites: *Neospora caninum* and *Toxoplasma gondii*. Canine adenovirus and herpesvirus were the most widely distributed pathogens, whereas *N. caninum* was relatively uncommon. Canine parvovirus and distemper had high annual variation, with western populations experiencing more frequent outbreaks than eastern populations. Seroprevalence of all infections increased as wolves aged, and denser wolf populations had a greater risk of exposure. Probability of exposure was positively correlated with human density, suggesting that dogs and synanthropic animals may be important pathogen reservoirs. Pathogen exposure did not appear to follow a latitudinal gradient, with the exception of *N. caninum*. Instead, clustered study areas were more similar: wolves from the Great Lakes region had lower odds of exposure to the viruses, but higher odds of exposure to *N. caninum* and *T. gondii*; the opposite was true for wolves from the central Rocky Mountains. Overall, mechanistic predictors were more informative of seroprevalence trends than latitude and longitude. Individual host characteristics as well as inherent features of ecosystems determined pathogen exposure risk on a large scale. This work emphasizes the importance of biogeographic wildlife surveillance, and we expound upon avenues of future research of cross-species transmission, spillover, and spatial variation in pathogen infection.

## Introduction

The prevalence and dynamics of infectious diseases can vary spatially across the distribution of their hosts depending on host demographics, host contact patterns, and pathogen survival. For example, Ferrari et al.^1^ showed how the cyclic dynamics of measles varies with human birth rate and seasonality. In a similar manner, Hudson et al.^2^ showed how the oscillations of red grouse (*Lagopus lagopus scotica*) abundance, driven by a caecal nematode, varied geographically according to the host growth rate and parasite transmission rate, and this drives longer cycle periods with increasing latitude. Pathogens that infect multiple host species may be more common at lower latitudes when this corresponds with increased numbers of host species or individuals. For example, parasites with complex life cycles that depend on the presence of intermediate hosts^3,4^ and seasonal aggregations, which vary with climate, can increase transmission and drive outbreaks^5^. In this paper we addressed the question: How does pathogen seroprevalence in gray wolves (*Canis lupus*) vary across North America and does geography provide a suitable proxy?

Generally, human and wildlife pathogen pressures (e.g., parasite burden, richness, prevalence) decline as latitude increases^6–11^ (i.e., towards the poles). Latitude is a proxy for other variables that predictably vary over space and affect pathogen transmission, which may be a function of pathogen survival or host density. For example, latitude can be used to describe the climate envelope for chytrid fungus, where higher latitudes (e.g., cooler temperatures, higher rainfall) are more optimal for fungal survival than lower latitudes. Consequently, chytrid infection intensity is significantly higher at higher latitudes^12^. Understanding the mechanisms driving transmission provides a deeper understanding of host–pathogen dynamics but requires detailed datasets that are often challenging to collect. Here, we assess how well geography alone can explain the observed variation in seroprevalence, and contrast this with variables that may confer a mechanistic understanding of pathogen exposure at individual and population levels, such as wolf and human densities, wolf age, sex, coat color, pack size, or habitat quality (Table 2).

**Figure 1:**
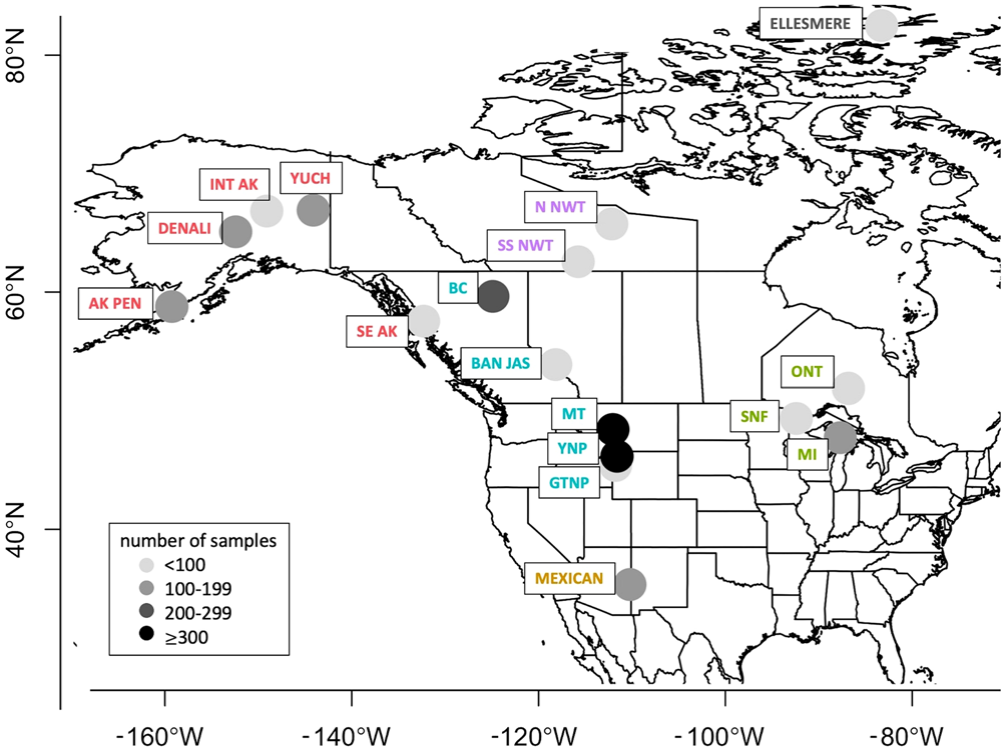
A map^38^ depicting where wolves were sampled across North America for pathogen and parasite testing, and relative sample size from each study area is shown in shades of gray (increasingly dark gray = increasing sample size)^39^. Each study area is identified as follows: Alaska Peninsula (AK PEN), Denali National Park (DENALI), central-eastern Alaska (INT AK), Yukon-Charley Rivers National Preserve (YUCH), Ellesmere Island (ELLESMERE), North Slave Northwest Territories (N NWT), South Slave Northwest Territories (SS NWT), British Columbia (BC), southeastern Alaska (SE AK), Banff and Jasper National Parks (BAN JAS), Montana (MT), Yellowstone National Park (YNP), Grand Teton National Park (GTNP), Mexican wolves (MEXICAN), Ontario (ONT), Superior National Forest (SNF), and the Upper Peninsula of Michigan (MI). Study area labels were colored by region: High Arctic (gray), Subarctic (purple), Alaska (red), central Rocky Mountains (turquoise), Great Lakes (green), and Mexican (gold), and displayed as a circle with a 200-km radius.

In North America, wolves suffer from both enzootic and epizootic pathogens that can result in chronic disease or acute outbreaks, causing morbidity, mortality, and reduced recruitment^13–16^. Patterns of seroprevalence across wolf populations have not been comprehensively explored, but individual studies have shown notable differences in seroprevalence. For instance, *Neospora caninum* antibodies were not detected in any wolves sampled from the Alaska Peninsula^17^, while 66% of adult wolves in northeastern Minnesota were seropositive^18^. This has constrained our understanding about what pathogens we can expect wolves to be exposed to and at what frequency. To investigate the drivers of pathogen exposure, we compiled a serological dataset of North American wolves spanning 17 study areas across 80° of longitude, from the Alaska Peninsula in the west to Ontario in the east, and 50° of latitude, from Ellesmere Island in the north to Arizona and New Mexico in the south (Fig. 1). Wolf sera were tested for antibodies to four viruses: canine adenovirus-1 (i.e., adenovirus), canine parvovirus-2 (i.e., parvovirus), canine distemper virus (i.e., distemper), canine herpesvirus (i.e., herpesvirus), and two protozoa: *Neospora caninum*, and *Toxoplasma gondii* (Table 1).

**Table 1:** A list of wolf pathogens that were examined for populations sampled across North America (Fig. 1) and their characteristics^37^. ‘Alternative hosts’ refers to hosts other than wolves that occur within the study areas that we expect to be important in transmitting pathogens to wolves. ‘Population consequences’ describes the known or expected severity of these pathogen infections on wolf population size or growth rate (minimal, moderate, severe).

Pathogen	Transmission route	Alternative Hosts	Symptoms / effects	Population consequences
Canine adenovirus	Direct via respiratory secretions; fecal–oral	None	Fever, liver inflammation	Mild
Canine distemper virus	Direct via respiratory droplets/secretions; airborne	Carnivores—grizzly (*Ursus arctos*) and black bears (*Ursus americanus*), cougars (*Puma concolor*), lynx (*Lynx canadensis*), coyotes (*Canis latrans*), raccoons (*Procyon lotor*), skunks (*Mephitis mephitis*), domestic dogs (*Canis lupus familiaris*)	Enamel hypoplasia, seizures, death—mostly affects pups or naive, immunocompromised adults	Severe (albeit acute)
Canine herpesvirus	Vertical; sexual; direct via respiratory droplets/ secretions	None	Adult females: abortion; pups: lethargy, sudden death	Mild
Canine parvovirus	Fecal–oral	Domestic dogs	Pups: diarrhea, lethargy, death	Moderate (but variable)
*Neospora caninum*	Ingesting infected tissue (definitive) or oocysts (intermediate); vertical	Intermediate: Ungulates Definitive: canids—coyotes, foxes (*Vulpes vulpes*)	Muscle weakness, tremors, loss of coordination	Mild
*Toxoplasma gondii*	Ingesting infected tissue or oocysts; vertical	Intermediate: Warm-blooded animals Definitive: felids—cougars, lynx, bobcat (*Lynx rufus*), domestic cats (*Felis catus*)	Increased aggression and risk-taking	Mild

**Figure 2:**
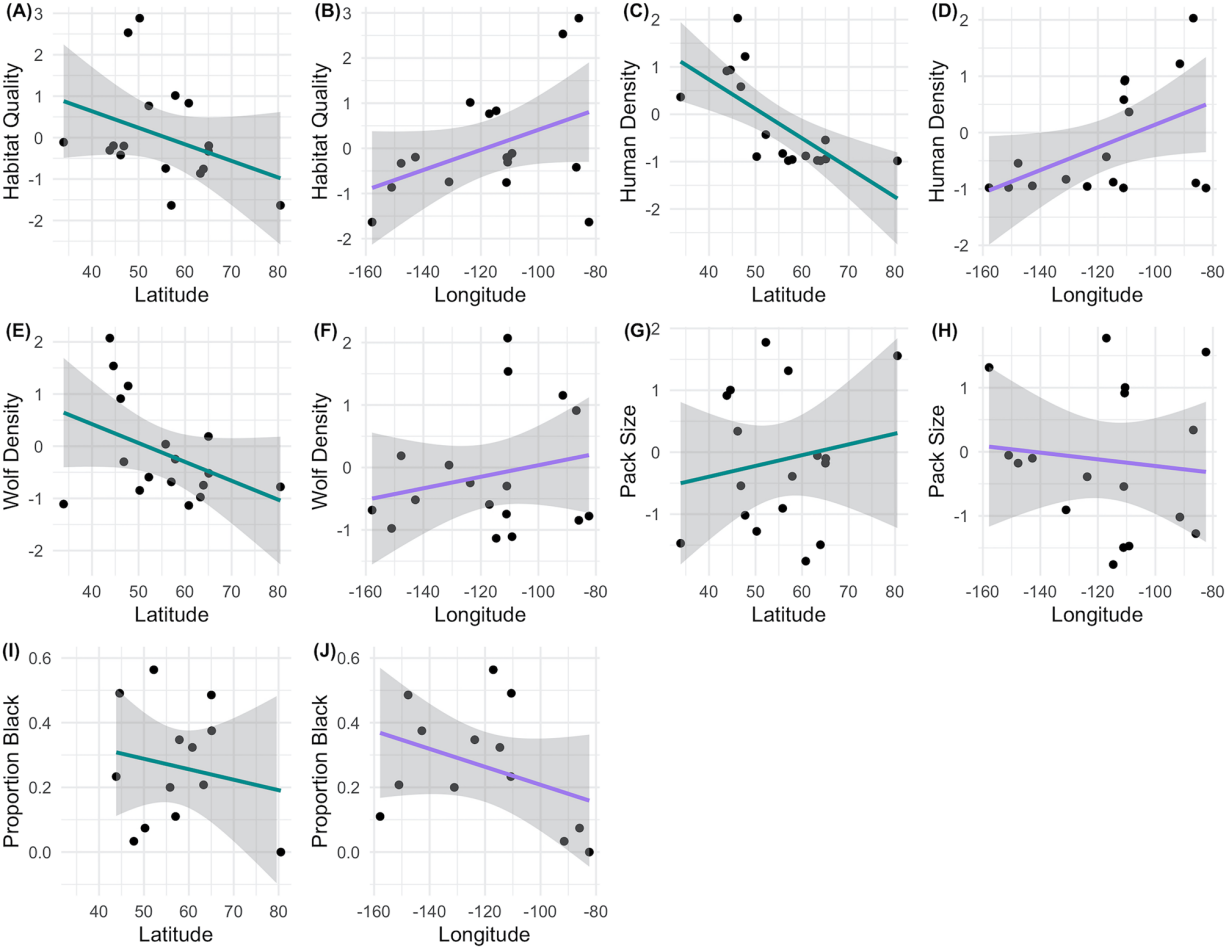
The effect of latitude (teal lines) and longitude (purple lines) on selected standardized, continuous predictor variables that were included in the generalized linear mixed models^39^: (**A**,**B**) habitat quality, (**C**,**D**) human density, (**E**,**F**) wolf density, (**G**,**H**) pack size, as well as (**I**,**J**) proportion of black wolves (for **I**,**J**, note that MI, MT, and N NWT were removed due to lack of coat color data; MEXICAN was removed as *Canis lupus baileyi* does not present a black phenotype). Gray polygons are 95% confidence intervals around univariate regression lines.

For directly transmitted pathogens (e.g., adenovirus, herpesvirus, parvovirus), contact rate (i.e., population density) determines transmission rates, and consequently pathogen seroprevalence and outbreak size^19^. Population density is also important for pathogens with environmental transmission (e.g., parvovirus) such that environmental reservoirs and contamination may accumulate more quickly at higher host densities^20^. The presence and population densities of sympatric reservoir hosts, including synanthropic animals, is also important for the dynamics of multi-host viruses (e.g., canine distemper, *T. gondii*)^21–26^, as well as parasites with intermediate hosts (e.g., *Neospora caninum*)^27^. Our large-scale dataset captures natural variation in human density, wolf density (e.g., population density, pack size, pack density), host presence (i.e., habitat quality), and primary prey, allowing us to examine their importance (Table 2, Fig. 2).

**Table 2:** A list of variables considered for inclusion in generalized linear mixed models predicting pathogen and parasite exposure. Variable descriptions and rationales or predictions are provided; a * indicates the variable was included in the final *complete model*, a ^+^ indicates the variable was included in the *geographic model.*

Variable name	Description	Rationale for inclusion/prediction
*Latitude*^+^	Latitude at study area centroid	Latitude may capture geographic variation in pathogen infections; we predicted that seroprevalence decreases as latitude increases.
*Longitude*^+^	Longitude at study area centroid	Longitude may capture geographic variation in pathogen infections.
Age class*^+^	Estimate of wolf age class: pup (< 1), subadult (1–2), and adult (≥ 3)	As individuals age, they have more time to be exposed to pathogens, thus older wolves will have higher seroprevalence. Age category is less error-prone than numerical age estimates.
Year*	Biological year, birth month = first month	Pathogen exposure may be predictable by year (i.e., endemics), or unpredictable (i.e., epidemics).
Study area*	Study area abbreviation	Study area may describe variation in pathogen exposure, not accounted for by other variables.
Habitat quality*	Index for habitat quality based on land cover type and topography	A continuous estimate of the habitat quality of the study area, this covariate considers habitat characteristics that carnivores, especially wolves, positively select. This is a proxy for the presence of sympatric carnivore hosts. Prediction: seroprevalence increases with habitat quality.
Human density*	Number of people/1000-km^2^	Provides information about how urban the area is, and thus the potential for contact between unvaccinated dogs or synanthropic species (e.g., rodents, coyotes, raccoons, skunks, cats) and wolves. Prediction: seroprevalence increases with human density.
Wolf density*	Number of wolves/1000-km^2^; mean annual density results in one estimate per study area	Population density is related to direct transmission rates and environmental contamination. Prediction: seroprevalence increases with wolf density.
Pack size*	Mean annual pack size; one estimate per study area	This tells us about the daily contacts of a wolf, which differs from contact rate at the population-level. Prediction: seroprevalence increases with pack size.
Sex*	Male or Female	There is evidence that males have higher pathogen prevalence than females across many taxa and pathogens—we predict males have higher seroprevalence.
Coat color*	Gray or Black	The locus that confers black coat color in wolves is linked to beta-defensin genes, which increases the responsiveness of the innate immune system. We assume gray = missing k-locus, black = presence of k-locus. Prediction: black wolves have higher seroprevalence.
*Age*	Estimate of wolf age; integer to two decimal places	As individuals age, they have more time to be exposed to pathogens, thus we predicted older wolves have higher seroprevalence.
*Social status*	Breeder or non-breeder	Breeders typically have higher stress levels and energetic demands than non-breeders, which we predict increases seroprevalence.
*Prey species*	Top two primary prey species	*N. caninum* or *T. gondii* may be more prevalent in different intermediate hosts. Prediction: seroprevalence is higher where white-tailed deer are a primary prey species.
*Pack membership*	Name of the pack the wolf was a member of when sampled	There may be heterogeneities in pathogen exposure based on pack membership.
*Pack density*	Number of packs/1000-km^2^; mean annual density results in one estimate per study area	Contact among wolves from different packs is likely influenced by the number of packs in the population. Prediction: seroprevalence increases with pack density.

In addition to larger scale processes, individual characteristics also play a role in wolf pathogen dynamics. North American wolves generally display two coat color phenotypes, black and gray, that vary latitudinally^28^. The black genotype is important for mounting immune responses^29^, and thus it has been hypothesized that the black color is maintained via selection from pathogen pressure^30,31^. This leads us to predict that black wolves are more likely to survive an exposure and test positive. Other individual traits, such as age and sex, may also influence pathogen exposure and should also be considered. Specifically, males tend to have higher pathogen prevalence than females due to physiology (e.g., sex and stress hormones) and behavior (e.g., contact patterns), and older individuals have had more time to be exposed to infectious diseases, thus tend to have elevated seroprevalence^32–36^.

In wildlife diseases literature, there is a lack of broad scale assessments in exposure trends that also include the animal’s ecology as mechanisms. We tested how well a suite of variables conferring mechanisms (Table 2) explained and predicted differences in probability of pathogen exposure across North American wolf populations, compared with latitude and longitude alone.

## Materials and methods

### Serology dataset

We aimed to compile a serological dataset from wolf populations across North America. We collaborated with wildlife professionals across the continent and attempted to obtain samples from a variety of ecosystems. While our dataset is expansive, there are gaps for two primary reasons: (1) wolves were not sampled or intensively studied in many areas, and (2) wolves do not occupy much of their historic North American range, especially in the south, thus they are absent from much of the United States. At a broad scale, our dataset is a fair representation of where wolves presently occur and are studied across North America.

All wolf samples analyzed for this study were previously collected by wildlife professionals within each study area. No wolf was captured or handled for the purposes of this study. Samples previously collected from live-captured wolves were handled and sampled according to the American Society of Mammalogists (Sikes et al. 2016), or similar guidelines, and approved by the appropriate institutions—see Supplementary Table S5 for specific approval and permits associated with samples included in our database. All samples from the northern Northwest Territories and about half from Ellesmere Island, Nunavut, were air dried, blood-soaked filter paper, and almost all other samples were serum collected from the cephalic or saphenous vein during live capture. We leveraged stored (i.e., frozen at − 20° to − 80° Celsius) samples for this study and, where needed, we coordinated with local wildlife professionals to ship serum to the Animal Health Diagnostic Center at Cornell University (Ithaca, NY, USA) where samples were screened for antibodies to the six pathogens of interest.

We screened wolf samples from 13 of the 17 study areas, and we used previously published serological results for the remaining 4 study areas (Supplementary Table S2). Previously published datasets included in our analyses were: Mexican^40^, Banff and Jasper National Parks^41^, Alaska Peninsula^17^, and a portion of the samples from Superior National Forest^18^. Here we discuss how samples were analyzed at the Animal Health Diagnostic Center at Cornell University, which comprised about 80% of our dataset (see Supplementary Table S3 for other testing information). Virus neutralization assays were performed to detect antibodies to canine adenovirus, distemper virus, and herpesvirus; hemagglutination inhibition assays were used for parvovirus; indirect fluorescent assays were used for *N. caninum*; enzyme-linked immunosorbent assay or monocyte activation tests were used for *T. gondii*. All assays provided titer values except for the indirect fluorescent and enzyme-linked immunosorbent assays, which provided a positive, negative, or suspect/equivocal result. Sample collection and test methods for the previously published samples were identical or equivalent to methods implemented for the other 13 study areas, thus are directly comparable (Supplementary Tables S2, S3).

The response variable in our models was a binary variable representing previous exposure (1), i.e., seropositive result, or not (0), i.e., seronegative result. A result was seropositive when the titer dilution was equal or greater than the standard titer cutoff provided by the assay manufacturer (Supplementary Table S3), or if the assay was positive/suspect (suspect comprised ~ 3% of the total dataset). As such, we assumed that serological assays were considered to be perfect, which is unlikely to be true. To address this, we assessed population seroprevalence using standard and conservative titer cutoffs; the standard cutoff is the lab-recommended value (Supplementary Table S3), and the conservative cutoff is one dilution above the standard cutoff. We found that pathogen prevalence was minimally affected by titer cutoff and we do not believe that this affected our results (Supplementary Fig. S1). Therefore, we present results using a standard titer cutoff specific to each assay and sample type. Note too that only individuals that survived an exposure were available to be sampled for serological analyses, thus lack of antibody detection may mean that the pathogen does not exist in that study area, or alternatively, that it caused high mortality locally and was not detected.

### Model construction

We constructed and analyzed models predicting the probability that a wolf was exposed to a given pathogen using R v3.6.3^39^. We tested how well geography (i.e., latitude and longitude) explained and predicted pathogen exposure compared with mechanistic predictor variables. Two models were constructed for each pathogen: a *complete model* and a *geographic model* (Eq. 1). The *geographic model*, which acted as a null/uninformative model, contained latitude and longitude, and controlled for the effect of age. The *complete model* contained selected predictor variables (Table 2). Both models included random effects (generalized linear mixed model, ‘GLMM’). Models were fit with a complementary-log–log link and a Bernoulli error distribution using the function *glmer* in the package *lme4*^42^. In the *complete model*, *year* and *study area* were both considered as random effects, where *year* was nested within *study area* because we posited that the effect of year differed within each study area. Nesting year within study area gave us a random effect for *study area* alone, as well as study area*year. *Study area* was the only random effect considered in the *geographic model*. The form of our GLMMs was:

1 $$Y_{ijk} = {\text{Bernoulli}}\left( {p_{ijk} } \right)$$

$$f\left( {p_{ijk} } \right) = \beta_{0} + \beta_{1} x_{1ijk} + \cdots + \beta_{n} x_{nijk} + \alpha_{j} + \gamma_{jk } + \varepsilon_{i}$$

$$\alpha_{j} \sim {\text{ Normal}}\left( {0, \, \sigma^{{2}} } \right)$$

$$\gamma_{jk} \sim {\text{ Normal}}\left( {0, \, \sigma^{{2}} } \right)$$

where *Y*_*ijk*_ is the seropositive result for the *n*_*jk*_ trial from the *i*th individual from the *j*th study area in year *k*; *p*_*ijk*_ is the probability of exposure from the *i*th individual from the *j*th study area in year *k*; *x*_*nijk*_ is the *i*th value of the *j*th study area in the *k*th year for the *n*th predictor; β_*n*_ are the estimated predictor coefficients; *α*_*j*_ is the study area-specific effect; *ɣ*_*jk*_ is the effect of year within that study area; *ε*_*i*_ is the remaining error in seropositivity. The *year* effects, including *ɣ*_*jk*_, did not appear in the *geographic model*. The link function (*f*) applied is the complementary-log–log.

All metadata were collected specifically for this project such that we determined our hypotheses *a priori*^43^ (Table 2, Supplementary Table S1). All variables considered were expected to influence pathogen exposure. Table 2 displays variables considered for inclusion in the models, descriptions, and rationales or predictions. Each sample was assumed to be unique, given that < 7% of the data were recollared wolves. If multiple age estimates were given (e.g., 3 or 4 years old), we randomly selected one age estimate. Some variables were removed prior to model building due to lack of sufficient data, including *pack membership*, *social status*, and *pack density* (Supplementary Table S4). *Prey species* was not included because primary prey species were too similar across study areas (e.g., a combination of elk, deer spp., moose, caribou), and after exploratory plotting, did not appear to provide additional information above *study area* and *habitat quality*. Prey species also are likely reflected in wolf density and pack size^44–47^. We included *age class* instead of *age* in our models because *age* was based on tooth wear and body size, and is an error-prone estimate especially for older ages^48^. We used coat color as a proxy for the presence of the K-locus allele, which is supported by Anderson et al.^28^ who found that > 98% of wolves from Yellowstone and western Canada classified as ‘black’ did indeed have the K-locus genotype.

We also considered *wolf density*, *pack size*, *human density*, *habitat quality*, and *sex* as potentially important predictors of pathogen exposure (Table 2). Wolves were counted in all study areas, including annual population counts and pack size estimates. These data were typically collected during aerial or ground tracking surveys in the winter. If more than one estimate was available per year within a study area, which was common for pack sizes, they were averaged to create one annual *wolf density* (number of wolves/1000-km^2^/year) and one annual mean *pack size* (mean number of wolves/pack/year) value per study area. To estimate *human density* and *habitat quality*, we first had to determine how large of an area should be considered, as most areas did not have clearly defined boundaries or isolated wolf populations. We considered a range of area sizes (radius 50-km to 300-km from study area centroids) and selected a 200-km radius because *human density* and *habitat quality* were less variable in comparison with small or large radii, and it is more congruent with wolf dispersal distance^49,50^. *Human density* was considered to be the number of people per 1000-km^2^^51^, and was used as a proxy for the presence of unvaccinated dogs and synanthropic animals^52^. *Habitat quality* was a proxy for the presence of carnivore hosts, and was a continuous variable calculated as the product of: percent forest cover^53^, percent area with slope ≤ 20° ^54^, and density of hard edges (e.g., cutblocks, pipeline cuts, forest edges; R package *landscapemetrics*^55^). These habitat characteristics were selected because they were considered positive predictors of carnivore presence, such as grizzly bears, lynx, bobcat, coyotes, with a focus on wolves^56–68^. While this proxy for carnivore presence is imperfect as carnivore distributions varied over our sampling distribution, and carnivores may select for different landscape features at different scales, it captures important features where wolves and other carnivores may interact, and therefore where cross-species pathogen transmission may occur. Finally, *sex* (male or female) was recorded during captures.

Before building the *complete model*, all variables were screened for collinearity using Spearman’s correlation coefficient (⍴). Human density and wolf density were highly correlated (⍴ = 0.62; Supplementary Fig. S3, S4) and thus were not included in the same model; however, as we were interested in the effects of both wolf and human density on pathogen dynamics, we ran the complete model both ways (i.e., with either wolf density or human density). All variables other than latitude and longitude were retained (i.e., correlation < 0.4). Latitude was highly correlated with human density (⍴ = − 0.79) and moderately correlated with wolf density (⍴ = − 0.36) and habitat quality (⍴ = − 0.33, Fig. 2, S3). Longitude was moderately correlated with human density (⍴ = 0.37), habitat quality (⍴ = 0.30), and proportion of black wolves (⍴ = − 0.33, Fig. 2, Supplementary Fig. S3). Our models were as follows (note that the divider between *year* and *study area* denotes the nested structure *study area* + study area*year):

#### Complete model

$$\begin{aligned} {\text{Probability}}\left( {{\text{exposure}}} \right) & \sim wolf \, density{\text{ or }}human \, density + \, habitat \, type \, \\ \quad & + \, pack \, size + \, age \, class \, + \, sex \, + \, color \, + \left( {study \, area \, | \, year} \right) \\ \end{aligned}$$

#### Geographic model

$${\text{Probability}}\left( {{\text{exposure}}} \right) \, \sim latitude + longitude + age \, class + study \, area$$

Continuous variables were standardized prior to model implementation (subtract the variable mean and divide by the standard deviation, Gelman and Hill^69^, Menard^70^). This centers all variables (mean = 0), and deviations from the mean are represented in standard deviations. Standardizing puts all continuous variables on the same scale, allowing for direct comparisons and simplifying interpretation. All models converged using the bobyqa optimizer.

### Model evaluation

Models were evaluated by root mean square error (RMSE) and area under the receiver-operator curve (AUC). RMSE and AUC provide different, important model evaluation. RMSE is a measure of model fit as it calculates the error between the observed data and the fitted model, whereas AUC provides a measure of the classification accuracy of the model; both criteria use model fixed effects. To calculate AUC, the false positive rate (1—specificity) is plotted against the true-positive rate (sensitivity); AUC = 0.5 indicates no discrimination, AUC > 0.5 indicates that the true positive rate is higher than the false-positive rate, and AUC > 0.8 indicates excellent discrimination^71^. We compared the testing set and training set RMSE and AUC using four-fold cross validation^72^ (see Supplementary Information for training and testing group information). Supplementary Figure S5 and Table S6 display the mean RMSE and AUC across the four datasets (training and testing) per pathogen and model.

Model fit assessments included: training and testing set RMSE and AUC estimates, pseudo-R2 values (calculated with fixed effects only), Maximum Likelihood estimator convergence, and p-values (i.e., hypothesis testing, Table 2). Predictor variables were considered statistically significant at an alpha value of ≤ 0.05. The geographic and complete models, parameter estimation, and their evaluations used all (non-missing) data.

## Results

### Dataset

We sampled 1839 wolves from 17 study areas to comprise the final dataset, with 134 wolves resampled, totaling 1973 rows of data. The mean number of samples per study area was 116 (95% confidence interval [CI] 90–142), ranging from 10 (SE AK) to 383 (YNP), but most study areas had between 50 and 150 samples. Most study areas were sampled for 10 years (95% CI 8.5–11.8, range = 2–25) and, on average, 12 wolves were sampled per year (95% CI 11–13). Collectively, study areas had a mean wolf density of 13 wolves/1000-km^2^ (95% CI 11–16, range = 3.4–34.0) and a mean of 6.3 wolves per pack (95% CI 5.8–6.8, range = 3.7–9.6). Habitat quality with respect to the presence of carnivore hosts was similar in most study areas, although a few populations stood out as low quality (AK PEN, Ellesmere Island), and others as high quality (ONT, SNF). Human density was more variable: some study areas had < 11 people/1000-km^2^ (AK PEN, Ellesmere, N NWT) and others had > 3000 people/1000-km^2^ (MI, SNF, YNP, GTNP, MT), with a mean of ~ 1600 people/1000-km^2^.

Most wolves sampled were adults (44%), and pups and subadults were equally sampled (28% each). Males and females were nearly equally sampled (52% and 48%, respectively), and there were more than twice as many gray wolves sampled (70%) as black wolves (30%). Some metadata were missing, in particular coat color, and missing information tended to be grouped by population (Supplementary Table S4).

**Figure 3:**
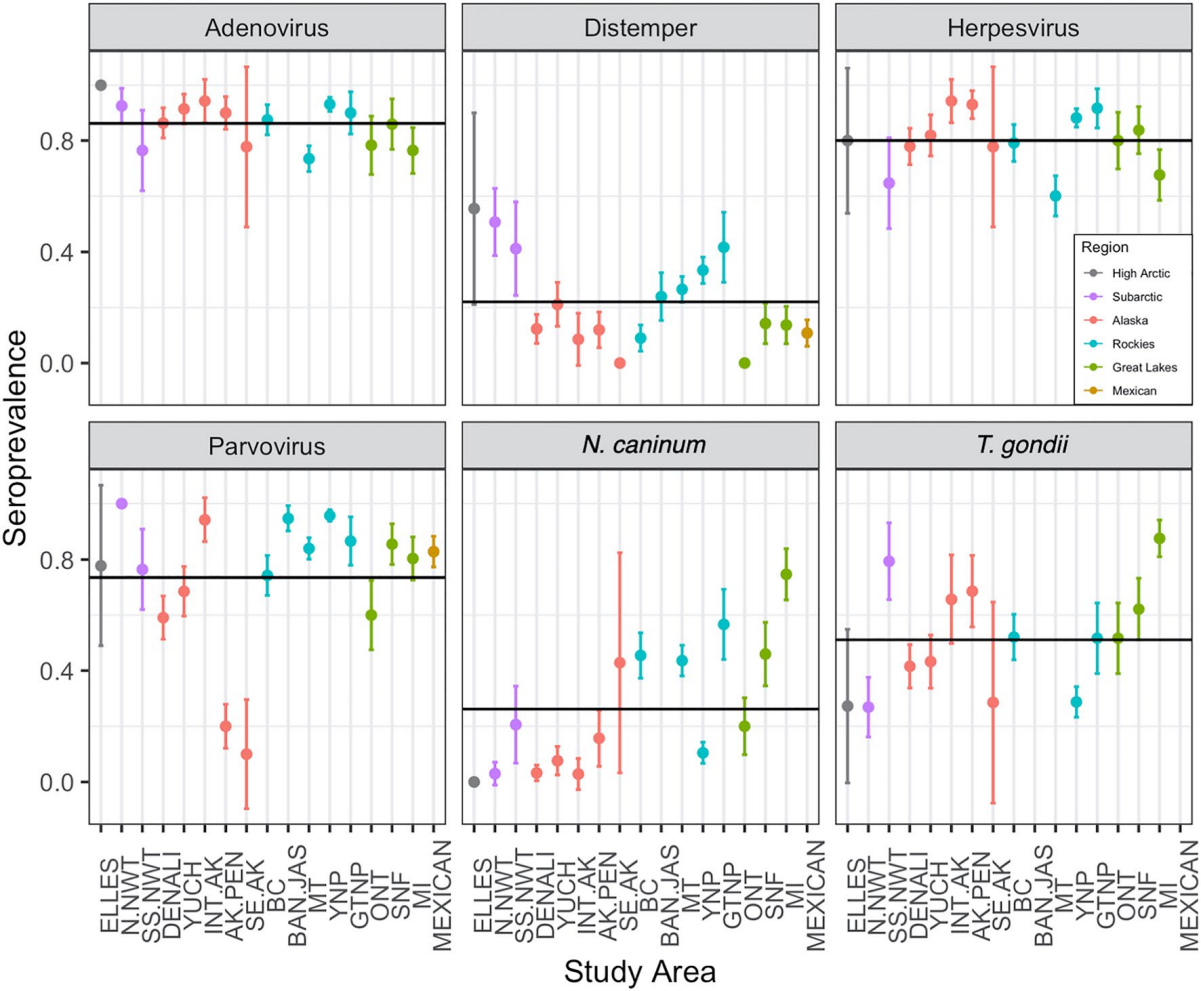
A panel plot displaying seroprevalence estimates, and 95% confidence intervals, for each pathogen tested from each study area^39^. Study areas are listed approximately north (left) to south (right) on the x-axis and grouped by region: High Arctic (gray), Subarctic (purple), Alaska (red), central Rocky Mountains (turquoise), Great Lakes (green), and Mexican (gold) (see Fig. 1 caption for study area abbreviations). The horizontal lines show the grand mean seroprevalence for each pathogen. Note that not all study areas were tested for each pathogen.

Adenovirus was the most widespread and prevalent pathogen (mean seroprevalence 86.2%, sd = 8.0%, range = 73.5–100%), followed by herpesvirus (mean seroprevalence 79.5%, sd = 11.3%, range = 57.1–94.3%, Fig. 3). *N. caninum* was the least common pathogen (mean seroprevalence 24.8%, sd = 24.4%, range = 0–74.7%, Fig. 3), and *T. gondii* was moderately prevalent across study areas (mean seroprevalence 51.5%, sd = 20.5%, range = 26.9–87.6%, Fig. 3). Distemper virus was relatively uncommon (mean seroprevalence 22.7%, sd = 18.0%, range = 0–55.6%, Fig. 3), but as an epizootic virus, overall seroprevalence is a poor representation of viral pressure or dynamics. We identified clear peaks in distemper seroprevalence in most populations that were sampled for at least five consecutive years (Supplementary Fig. S2). Evidence of exposure to parvovirus was the most variable (mean seroprevalence 73.8%, sd = 25.0%, range = 10.0–100%). Interestingly, parvovirus tended to be enzootic (e.g., BAN JAS, GTNP, MT, SNF, YNP) or common but variable (e.g., BC, DENALI, MEXICAN, MI, SS NWT, YUCH), but was uncommon in some study areas (e.g., AK PEN, SE AK), or patterns were unclear (e.g., INT AK, ELLES, N NWT, ONT, Fig. 3, Supplementary Fig. S2).

### Model results

**Figure 4:**
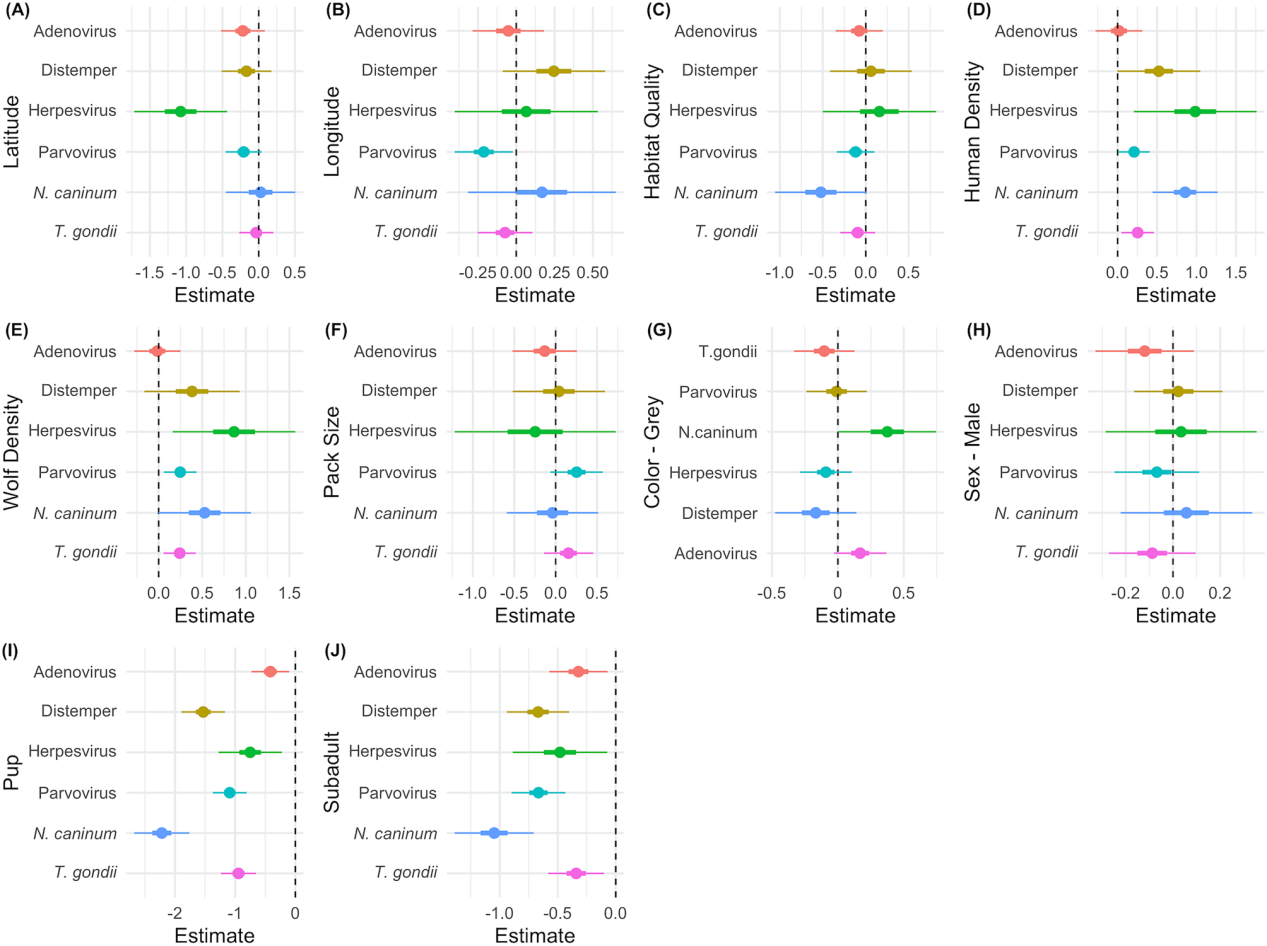
Coefficient estimates (log-odds) of the fixed effects in the (**A**,**B**) *geographic* and (**C**–**J**) *complete models* by pathogen (colors)^39^. Thick and thin lines are 50% and 95% confidence intervals, respectively. Categorical variables are interpreted as the effect of: (**G**) gray wolves with respect to black, (**H**) males with respect to females, and (**I**) pups and (**J**) subadults with respect to adults.

The coefficient estimates (β) for latitude were negative for all pathogens except adenovirus and distemper where β ~ 0. However, latitude was only a statistically significant predictor of *N. caninum* exposure such that the probability of exposure to *N. caninum* decreased appreciably as latitude increased across North America (i.e., northward, Fig. 4A, Supplementary Fig. S14, Table S7). The effect of longitude was variable: the coefficient estimates for longitude were negative for adenovirus and herpesvirus, positive for parvovirus and *N. caninum*, and approximately zero for distemper and *T. gondii* (Fig. 4B). Longitude was only a statistically significant predictor of herpesvirus exposure such that the probability of exposure to herpesvirus decreased as longitude increased across North America (i.e., eastward)—although statistically significant, the effect size of longitude on herpesvirus was relatively small as herpesvirus was common in our sampled study areas (mean seroprevalence ~ 80% Fig. 3, S10). Pseudo-R^2^ values (Cragg-Uhler approximation, see SI) were lower for *geographic models* compared with *complete models* for the adenovirus, distemper, and herpesvirus; *geographic model* pseudo-R^2^ was higher for the *N. caninum complete model*; pseudo-R^2^ values were equal for both models for parvovirus and *T. gondii*. In general, the selected predictor variables accounted for a larger proportion of the variation in exposure than latitude and longitude.

The effect of *habitat quality* on pathogen exposure varied and tended to be small (*β* < 0 adenovirus, distemper, herpesvirus, *T. gondii*; *β* > 0 parvovirus, *N. caninum*); *habitat quality* was only considered a statistically significant predictor of canine distemper (Fig. 4C). Increasing *human density* was significantly and positively related to the probability of pathogen exposure for all pathogens (*β* > 0, p ≤ 0.05 except *T. gondii*; Fig. 4D). *Human density* had large effects on distemper, parvovirus, and *N. caninum*—for example, the probability a wolf was seropositive for distemper increased 68% over the *human density* range assessed (Supplementary Fig. S9). Similarly, *wolf density* was positively related to the probability of pathogen exposure for all pathogens (β > 0), except *T. gondii* (β ~ 0), and was a statistically significant predictor of pathogen exposure for pathogens except parvovirus and *T. gondii* (Fig. 4E). The effect of *pack size* on probability of exposure was variable (β < 0 *N. caninum, T. gondii;* β > 0 adenovirus, herpesvirus, parvovirus; β ~ 0 distemper), but these effects were small and statistically insignificant (Fig. 4F). Contrary to our predictions, probability of pathogen exposure was invariant to *coat color* and *sex* such that effect sizes were small and statistically insignificant (Fig. 4H,G); the exception was that gray wolves had a slightly higher probability of exposure to *N. caninum* than black wolves. As expected, seroprevalence increased with age for all pathogens (Fig. 4I,J). See SI *Model Results* (Supplementary Table S7) for additional modeling outputs.

**Figure 5:**
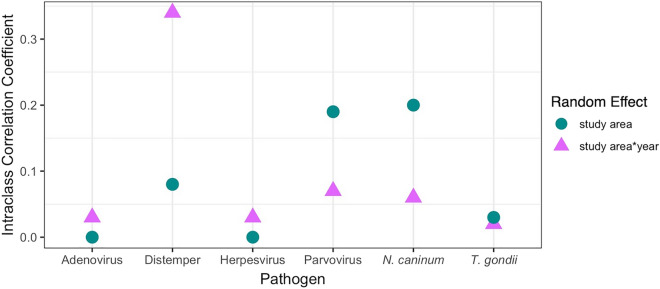
Intraclass correlation coefficient values for the random effects from the *complete models*: *study area* (teal circles) and study area*year (purple triangles)^39^*.*

We performed a four-fold cross validation whereby 13 study areas were used as the training set and the remaining four study areas were used as the testing set (Supplementary Fig. S5, Table S6). Testing set RMSE values were higher than RMSE values from models built using the training set, indicating that predictive power was weaker than explanatory power, as expected^72^. This also suggests that model fit was not highly dependent on which study areas were used in the training or testing sets. *Geographic models* had marginally higher RMSE and lower AUC than *complete models*, indicating slightly poorer fit and classification power. Regardless of model, exposure to some pathogens was better explained than others (e.g., poorest fit for *T. gondii*, best fit for adenovirus and herpesvirus). RMSE values were fairly high across all models, meaning that there was a significant amount of variation in pathogen exposure that was unaccounted for—especially *T. gondii*. This was also evident in that random effects accounted for a notable portion of the variation in pathogen exposure (Fig. 5), and pseudo-R^2^ values were fairly low (< 0.4).

Models had moderate power to correctly classify an individual as positive or negative for pathogen exposure (mean training set AUC = 0.69, mean testing set AUC = 0.67). For pathogens other than *T. gondii*, AUC dropped, on average, 2–4% from training to testing sets when evaluating the same pathogen; the training set AUC was, on average, about 4% higher using *complete models* compared with *geographic models*, and the testing set AUC improved 2.3%. *Complete models*, therefore, provided modest improvements to the *geographic models*.

### Random effects

Random effects (*study area* and study area*year) accounted for a notable portion of the deviance in exposure status (range = 0–33%, mean = 9%). We explicitly compared the random effects by calculating intraclass correlation coefficient, which describes the proportion of variance in exposure probability that the grouping accounts for (i.e., *study area* or study area*year, Fig. 5). The effect of *study area* and study area*year differed by pathogen, but we can generalize that: (1) *study area* was most important for *N. caninum* and parvovirus, (2) study area*year was most important for distemper virus due to its epizootic nature, (3) random effects were not very important for pathogens that were universally prevalent (e.g., adenovirus and herpesvirus), and (4) both *study area* and study area*year did not account for very much variation in *T. gondii* exposure, potentially because it was fairly common in all sampled study areas (Fig. 3). For example, in Yellowstone, the odds of distemper exposure differed up to tenfold among years (Supplementary Fig. S18A), whereas *T. gondii* exposure was stable (Supplementary Fig. S18B). Parvovirus exposure was most variable across study areas, but there was still some fluctuation within study areas. In other words, parasite exposure was more dependent on spatial dynamics whereas epizootic viruses were more dependent on temporal dynamics.

We can also draw conclusions from assessing the *study area* random intercepts, which provides a comparison to the baseline, or grand mean, probability of pathogen exposure across North American wolf populations (Supplementary Fig. S19, S20). The probability of contracting *N. caninum* increased significantly from north to south; *T. gondii* was more variable, and wolves from Michigan and South Slave Northwest Territories had particularly high odds of exposure. Epizootic viruses (i.e., parvovirus and distemper) had less predictable latitudinal trends, but Great Lakes and Alaska wolves generally had lower odds of exposure. Wolves in the central Rocky Mountains (except British Columbia) were more likely to be seropositive for both parvovirus and distemper, and South Slave Northwest Territories and Mexican wolves also had higher probability of distemper exposure. Adenovirus and herpesvirus antibodies were highly prevalent across all study areas sampled (often > 75% seroprevalence, Fig. 3), thus all intercept estimates hovered around the grand mean.

## Discussion

Spatial variation in pathogen infections in wide-ranging hosts have been described by latitudinal gradients^5–7,11,12,73,74^. While latitude may predict pathogen dynamics, it does not elucidate the underlying mechanisms. This is largely because necessary datasets to assess mechanisms of exposure are difficult to acquire across a species’ geographic range. Our objectives were to describe the spatial variation in seroprevalence of gray wolves spanning the North American continent, identify which variables best predict pathogen exposure, and expand our understanding about the mechanisms driving pathogen dynamics. Specifically, we focused on the effect of latitude as a primary driver of spatial variation in seroprevalence. To this end, we compiled an expansive serological dataset that captures the natural variation in pathogen seroprevalence as well as variables at the ecosystem, population, and individual scales (Figs. 1, 2). The effect size of latitude was greatest for *N. caninum* exposure, and compared with the other study areas, *N. caninum* seroprevalence trends most closely tracked latitude (Figs. 3, 4). Study areas in close proximity were more likely to be similar; for example, Great Lakes wolves had a lower probability of exposure for distemper and parvovirus, whereas wolves in the Arctic and central Rocky Mountains had higher probabilities. Our results highlight that individual host characteristics, as well as inherent features of ecosystems, determine pathogen exposure risk.

Human density was correlated with an increased probability of exposure of the four viruses of interest and *N. caninum*. Human density may be a proxy for density of unvaccinated dogs or synanthropic animals that act as reservoirs for infectious diseases that spill over into wolves^52^. Domestic dogs in Africa are the primary reservoir for canine distemper and rabies, and are responsible for major epizootics from these diseases in other wildlife species following spillover^23,25,75,76^. Across North America, we expect that dogs and synanthropic wildlife (e.g., raccoons, skunks, rodents) are important reservoirs for transmitting canine distemper, parvovirus, and *T. gondii*. Once spillover has occurred, wolf contact rate (i.e., density) must be high enough for wolf-wolf transmission. This might explain why we observed higher distemper and parvovirus seroprevalence in populations with both high human and wolf densities: Yellowstone, Grand Teton, and Banff and Jasper National Parks. However, dog and human densities may not always covary—for instance, dog density is high in areas where dog sledding is popular (Alaska, Northwest Territories), but human density is low. Additionally, some populations did not follow this rule and warrant further investigation, such as the South Slave region of the Northwest Territories that had low wolf, human, and carnivore density, yet high distemper seroprevalence, and Mexican wolves that displayed relatively high seroprevalence and risk of exposure despite low wolf density.

We predicted that study areas with larger pack sizes would have higher pathogen seroprevalence, which has been demonstrated in primates^77,78^. On the other hand, larger packs may aid in individual recovery from non-immunizing, chronic infections such as *N. caninum*, similar to wolves with sarcoptic mange^15^. However, mean pack size was not an important predictor of exposure to any pathogen in our models. We suggest that any effect of pack size on exposure risk may have been obscured by averaging across groups.

We predicted that better quality habitats would be more speciose and thus multi-host pathogens would occur at higher prevalences^79–81^. Interestingly, our results demonstrate a weak negative correlation between habitat quality and exposure probability. Our habitat quality index may have been a poor proxy for habitat quality, or not representative of quality habitat for other competent hosts. In reality, understanding the dynamics of multi-host pathogens requires knowledge about host contact rates, transmission, and pathogen reservoirs.

We expected black wolves to have a higher probability of pathogen exposure, in particular, canine distemper virus. Mechanistically this could occur because black wolves have improved immune responses to respiratory pathogens, and heterozygote black wolves have higher survival rates than their gray counterparts, especially in years of canine distemper virus^29–31,81,82^. Thus if black wolves survived pathogen infections at a higher rate, there would be more seropositive black wolves than gray wolves. We found that wolves in western study areas experienced more frequent distemper outbreaks and had a high proportion of black wolves (> 30%, Fig. 2, Supplementary Fig. S2). Wolves in the Great Lakes region experienced reduced pressure from distemper, and accordingly, had a much lower proportion of black wolves (< 5%, Fig. 2, Supplementary Fig. S2). However, wolf phenotype in the Great Lakes may also be influenced by historical hybridization with eastern wolves (*C. l. lycaon*)^83^. Still, coat color was not a significant predictor of exposure to any pathogen except *N. caninum*. This does not preclude a relationship between coat color and pathogen infections, and potentially suggests that pathogen pressure may predict coat color, which would reverse the response and predictor variable compared to our GLMMs.

*Neospora caninum* was the only pathogen we investigated that showed a strong latitudinal gradient in risk of pathogen exposure (Fig. 4) and mean seroprevalence (Fig. 3). We postulate that this corresponds to the proportion of white-tailed deer (*Odocoileus virginianus*) in the local wolf diets. The *N. caninum* cervid-canid lifecycle is well established^84^, and white-tailed deer are considered to be the *N. caninum* reservoir^27,85^. *N. caninum* has been detected at low levels in North American caribou (*Rangifer tarandus*), elk (*Cervus canadensis*), bison (*Bison bison*), mule deer (*Odocoileus hemionus*), and moose (*Alces alces*)^27,86–89^, but robust and widespread sampling is generally lacking. Based on our findings, it appears that the probability of *N. caninum* exposure varies with white-tailed deer consumption: higher in the Great Lakes region (mean seroprevalence 47%) where wolves primarily consume white-tailed deer and moose, moderate in the central Rocky Mountains (mean seroprevalence 39%) where wolves opportunistically consume deer, and uncommon in Alaska, the Northwest Territories, and Nunavut (mean seroprevalence 12%) where white-tailed deer do not occur^49,90–97^. This supports the notion that white-tailed deer are the natural hosts for *N. caninum*, although livestock consumption may also play a role, and both should also be evaluated such as adding diet data or deer/livestock density into future models.

The *complete models* provided modest improvements to the *geographic models* in terms of model fit and predictive power, indicating that mechanistic variables described a greater proportion of the observed variation in pathogen exposure than geography alone. More importantly, this provides a stronger interpretation of the drivers of pathogen exposure. However, serological data and corresponding host metadata are logistically challenging to collect and compile, thus our results also suggest that, for some host–pathogen systems, information from adjacent wolf populations may provide decent insight into pathogen dynamics.

## Conclusion

Elucidating the biogeographic patterns of pathogen exposure in a single host species across its distribution provides us with a deeper understanding of the mechanisms driving exposure, how these drivers predictably vary through space and time, and potential effects on host population dynamics or individual vital rates. We identified human density as a major driver of pathogen exposure at a continental scale. Anthropogenic environments create opportunities for aggregations of reservoir hosts and pathogen persistence, which in turn can affect wildlife—even wildlife that purposefully avoid human activity centers, like gray wolves^63,98^. Large-scale pathogen patterns have not been previously identified for the gray wolf, and here we show that regional rather than latitudinal patterns of seroprevalence were supported, with antibodies to viral pathogens more commonly identified among wolves in the Rocky Mountains whereas antibodies to parasites were more commonly identified among wolves in the Great Lakes region. This work builds upon previous studies and will hopefully serve as a catalyst for additional investigations into carnivore disease ecology, multi-host transmission dynamics, and biogeographic wildlife surveillance.

## Supplementary Information

Supplementary Information.

## Data Availability

All data and code are publicly available on the Dryad Repository 10.5061/dryad.5hqbzkh51.
