# Author Correction: Patterns and processes of pathogen exposure in gray wolves across North America

**DOI:** 10.1038/s41598-021-02031-6

**Published:** 2021-11-15

**Authors:** Ellen E. Brandell, Paul C. Cross, Meggan E. Craft, Douglas W. Smith, Edward J. Dubovi, Marie L. J. Gilbertson, Tyler Wheeldon, John A. Stephenson, Shannon Barber-Meyer, Bridget L. Borg, Mathew Sorum, Daniel R. Stahler, Allicia Kelly, Morgan Anderson, H. Dean Cluff, Daniel R. MacNulty, Dominique E. Watts, Gretchen H. Roffler, Helen Schwantje, Mark Hebblewhite, Kimberlee Beckmen, Heather Fenton, Peter J. Hudson

**Affiliations:** 1 Center for Infectious Disease Dynamics, Department of Biology, Huck Institutes of the Life Sciences, Pennsylvania State University, University Park, PA 16802 USA; 2 U.S. Geological Survey, Northern Rocky Mountain Science Center, Bozeman, MT 59715 USA; 3 Department of Ecology, Evolution, and Behavior, University of Minnesota, Saint Paul, MN 55108 USA; 4 Yellowstone Center for Resources, Wolf Project, P.O. Box 168, Yellowstone National Park, WY 82190 USA; 5 Animal Health Diagnostic Center, College of Veterinary Medicine, Cornell University, Ithaca, NY 14850 USA; 6 Department of Veterinary Population Medicine, University of Minnesota, Saint Paul, MN 55108 USA; 7 Ontario Ministry of Natural Resources and Forestry, Trent University, 2140 East Bank Drive, Peterborough, ON K9L 1Z8 Canada; 8 Grand Teton National Park, P.O. Drawer 170, Moose, WY 83012 USA; 9 U.S. Geological Survey, Northern Prairie Wildlife Research Center, 8711 37th St. SE, Jamestown, ND 58401 USA; 10 Denali National Park and Preserve, Central Alaska Inventory and Monitoring Network, P.O. Box 9, Denali Park, AK 99755 USA; 11 Yukon-Charley Rivers National Preserve, Central Alaska Inventory and Monitoring Network, 4175 Geist Road, Fairbanks, AK 99709 USA; 12 Department of Environment and Natural Resources, Government of the Northwest Territories, P.O. Box 900, Fort Smith, NT X0E 0P0 Canada; 13 British Columbia Ministry of Forests, Lands, Natural Resource Operations and Rural Development, 2000 South Ospika Blvd., Prince George, BC V2N 4W5 Canada; 14 Environment and Natural Resources, Government of the Northwest Territories, North Slave Region, NT X1A 2P9 Canada; 15 Department of Wildland Resources, Utah State University, Logan, UT 84322 USA; 16 U.S. Fish and Wildlife Service, Kenai National Wildlife Refuge, P.O. 2139, Soldotna, AK 99669 USA; 17 Division of Wildlife Conservation, Alaska Department of Fish and Game, 802 3rd Street, Douglas, AK 99824 USA; 18 Wildlife and Habitat Branch, British Columbia Ministry of Forests, Lands, Natural Resource Operations and Rural Development, 2080 Labieux Road, Nanaimo, BC V9T 6J9 Canada; 19 Wildlife Biology Program, Department of Ecosystem and Conservation Sciences, Franke College of Forestry and Conservation, University of Montana, Missoula, MT 59812 USA; 20 Division of Wildlife Conservation, Dept of Fish and Game, 1300 College Road, Fairbanks, AK 99701 USA; 21 Ross University School of Veterinary Medicine, Basseterre, West Indies KN-03 St. Kitts and Nevis

Correction to: *Scientific Reports* 10.1038/s41598-021-81192-w, published online 12 February 2021

Heather Fenton was omitted from the author list in the original version of this Article.

The Acknowledgements section now reads:

“We thank the wildlife professionals that contributed wolf sera, serology data, and metadata to this project: Erin Stahler, L. David Mech, Dean Beyer, Erin Largent, Susan Dicks, John Oakleaf, the Mexican Wolf Project, Lindsey Dreese, Brent Patterson, Emily Almberg, Montana Fish Wildlife and Parks, Shari Willmott, and the countless unlisted biologists that collected these samples and data through the decades. Financial support includes: E.E.B. and P.J.H. endowment from Verne Willaman; E.E.B. and P.C.C. U.S. Geological Survey (Grant G17AC00427); D.W.S., D.R.S., and D.R.M. NSF LTREB grant DEB–1245373 and many donors to Yellowstone Forever, especially Annie and Bob Graham and Valerie Gates; M.H. Parks Canada and NSF LTREB award 1556248; G.R. Pittman-Robertson Federal Aid in Wildlife Restoration Program and the State of Alaska general funds; K.B. Federal Aid in Support of Wildlife Restoration and Alaska Dept. of Fish and Game; M.A. and H.S. British Columbia Ministry of Forests, Lands, Natural Resource Operations and Rural Development, Habitat Conservation Trust Foundation, Forest Enhancement Society of British Columbia; D.R.M and M.A. Polar Continental Shelf Project and National Geographic Society; M.L.J.G. the Office of the Director, National Institutes of Health under award number NIH T32OD010993; T.W. Ontario Ministry of Natural Resources and Forestry; B.L.B. National Park Service; A.K. GNWT Environmental Stewardship Fund. The content is solely the responsibility of the authors and does not necessarily represent the official views of the National Institutes of Health. Any use of trade, product, or firm names is for descriptive purposes only and does not imply endorsement by the U.S. Government.”

The original Article and accompanying Supplementary Information file have been corrected.

