# Supplementary material for: Patterns and processes of pathogen exposure in gray wolves across North America
Source: Sci Rep. 2021 Feb 12;11:3722. doi: 10.1038/s41598-021-81192-w (PMC7881161; doi:10.1038/s41598-021-81192-w)
Supplement: Supplementary file 1 — Supplementary Information. [file 41598_2021_81192_MOESM1_ESM.pdf]

Supplementary Information: Patterns and processes of pathogen exposure in gray wolves across North America

Ellen E. Brandell, Paul C. Cross, Meggan E. Craft, Douglas W. Smith, Edward J. Dubovi, Marie L.J. Gilbertson, Tyler Wheeldon, John A. Stephenson, Shannon Barber-Meyer, Bridget L. Borg, Mathew Sorum, Daniel R. Stahler, Allicia Kelly, Morgan Anderson, H. Dean Cluff, Daniel R. MacNulty, Dominique E. Watts, Gretchen H. Roffler, Helen Schwantje, Mark Hebblewhite, Kimberlee Beckmen, Heather Fenton, Peter J. Hudson

Serology dataset
Below is a description of each column included in the final dataset.

pop: ‘population’ or study area ID = Alaska Peninsula (AK PEN), Denali National Park (DENALI), central-eastern Alaska (INT AK), Yukon-Charley National Preserve (YUCH), Ellesmere Island (ELLESMERE), North Slave Northwest Territories (N NWT), South Slave Northwest Territories (SS NWT), British Columbia (BC), southeastern Alaska (SE AK), Banff & Jasper National Parks (BAN JAS), Montana (MT), Yellowstone National Park (YNP), Grand Teton National Park (GTNP), Mexican wolves (MEXICAN), Ontario (ONT), Superior National Forest (SNF), and the Upper Peninsula of Michigan (MI).
year: biological year; year starts according to the birth month in each study area; April = British Columbia/Banff/Jasper National Parks and south, May = SE Alaska and north).
age.cat: age category as pup [0,1), subadult [1,3), and adult [3+].
sex: F female, M male, or NA.
color: B black, G gray, or NA.
lat: latitude at study area centroid.
long: longitude at study area centroid.
habitat*: a proxy for the presence of carnivore hosts, and was a continuous variable calculated as the product of: percent forest cover[1], percent area with slope ≤20º [2], and density of hard edges (e.g., cutblocks, pipeline cuts, forest edges; R package *landscapemetrics*[3]). These habitat characteristics were selected because they were considered positive predictors of carnivore presence, such as grizzly bears, lynx, bobcat, coyotes, with a focus on wolves [4–16]. While this proxy for carnivore presence is imperfect as carnivore distributions varied over our sampling distribution, and carnivores may select for different landscape features at different scales, it captures important features where wolves and other carnivores may interact, and therefore where cross-species pathogen transmission may occur.
human*: the number of people per 1000-km^2 [17], and was used as a proxy for the presence of unvaccinated dogs and synanthropic animals[18].
pop.density: average population density (wolves/1000-km^2/year). This was calculated as a basic average across years, but for populations with >1 density estimate per year, density was first averaged by year, resulting in one density estimate per year.
pack.size: average annual pack size (mean number of wolves/pack/year); pack sizes were averaged within a year, then averaged across years to provide one average annual pack size estimate per study area.
standard.habitat: standardized **habitat** column.

standard.human: standardized **human** column.
standard.pop: standardized **pop.density** column.
standard.packsize: standardized **pack.size** column.
standard.latitude: standardized **lat** column.
standard longitude: standardized **long** column.
***pathogen*.binary:** 0 negative, 1 positive using the titer cutoffs in Table S3.
pathogen:

cav = canine adenovirus
chv = canine herpesvirus
cdv = canine distemper virus
cpv = canine parvovirus-2
neo = *Neospora caninum*
toxo = *Toxoplasma gondii*

*Study areas are assumed to be the same size: a circle with radius 200-km^2 (total area = 125664-km^2) based on typical wolf territory size and dispersal distance. Centroids were the approximate center of samples, verified by resident expert biologists.

Table S1. The metadata reference for each study area.

Study Area	Data Reference
AK PEN	Watts, D.E. and Benson, A.M., 2016. Prevalence of antibodies for selected canine pathogens among wolves (Canis lupus) from the Alaska Peninsula, USA. Journal of wildlife diseases, 52(3), pp.506-515.
BAN JAS	Hebblewhite, M., P. C. Paquet, D. H. Pletscher, R. B. Lessard, and C. J. Callaghan. 2003. Development and application of a ratio-estimator to estimate wolf killing rates and variance in a multiple prey system. Wildlife Society Bulletin 31:933-946.
BC	BC Ministry of Forests, Lands, Natural Resource Operations, and Rural Development
DENALI	Denali Wolf Project, Denali National Park and Preserve, Denali Park, AK, USA.
ELLESMERE	Anderson, M., D. MacNulty, H. D. Cluff, and L. D. Mech. 2019. High Arctic wolf ecology: Final report 2014-2018. Submitted to Wildlife Research Section, Government of Nunavut, Igloolik, NU. 50 pp
GTNP	Grand Teton National Park, USA
INT AK	Alaska Department of Fish & Game, Division of Wildlife Conservation, Fairbanks, AK, USA
MEXICAN	Mexican Wolf Project, USFWS. Arizona Game and Fish Department.
MI	Michigan Department of Natural Resources
MT	Montana Fish, Wildlife, & Parks
N NWT	Department of Environment and Natural Resources, Government of the Northwest Territories
ONT	Ontario Ministry of Natural Resources and Forestry, Wildlife Research & Monitoring Section, Peterborough, ON; Holloway, J. 2009. Size dependent resource use of a hybrid wolf (C. lupus X C. lycaon) population in northeast Ontario. (Msc thesis); Anderson, M. 2012. Wolf responses to spatial variation in moose density in northern Ontario. (Msc Thesis).
SE AK	Alaska Department of Fish & Game; Roffler, G.H., Waite, J.N., Pilgrim, K.L., Zarn, K.E. and Schwartz, M.K., 2019. Estimating abundance of a cryptic social carnivore using spatially explicit capture–recapture. Wildlife Society Bulletin, 43(1), pp.31-41.
SNF	U.S. Geological Survey, MN Wolf and Deer Project, Northern Prairie Wildlife Research Center, Jamestown, ND, USA
SS NWT	Department of Environment and Natural Resources, Government of the Northwest Territories
YNP	Yellowstone Wolf Project, Yellowstone National Park, USA
YUCH	Schmidt, J.H., Burch, J.W. and MacCluskie, M.C., 2017. Effects of control on the dynamics of an adjacent protected wolf population in interior Alaska. Wildlife Monographs, 198(1), pp.1-30.

Table S2. The lab, sample type, and pathogens tested (CAV adenovirus, CDV distemper, CHV herpesvirus, CPV parvovirus, NEO *N. caninum*, TOXO *T. gondii*) from each study area. The reference for previously published serological data is included, otherwise samples were tested for the purposes of this study. If relevant, the type of TOXO test is included (see Table S3).

Study Area	Lab	Sample	Pathogens	Reference
AK.PEN	WADDL	serum	CAV, CDV, CHV, CPV, NEO (IFA), TOXO (IFA)	Watts, D.E. and Benson, A.M., 2016. Prevalence of antibodies for selected canine pathogens among wolves (Canis lupus) from the Alaska Peninsula, USA. Journal of Wildlife Diseases, 52(3), pp.506-515.
BAN JAS	WCVM	serum	CDV, CPV	Nelson, B., Hebblewhite, M., Ezenwa, V., Shury, T., Merrill, E.H., Paquet, P.C., Schmiegelow, F., Seip, D., Skinner, G. and Webb, N., 2012. Prevalence of antibodies to canine parvovirus and distemper virus in wolves in the Canadian Rocky Mountains. Journal of Wildlife Diseases, 48(1), pp.68-76.
BC	Cornell	serum	CAV, CDV, CHV, CPV, NEO, TOXO (MAT)	This paper.
DENALI	Cornell	serum	CAV, CDV, CHV, CPV, NEO, TOXO (MAT)	This paper.
ELLESMERE	Cornell*	serum, strips	CAV, CDV, CHV, CPV, NEO, TOXO (MAT)	This paper.
GTNP	Cornell	serum	CAV, CDV, CHV, CPV, NEO, TOXO (MAT)	This paper.
INT AK	Cornell	serum	CAV, CDV, CHV, CPV, NEO, TOXO (MAT)	This paper.
MEXICAN	NMVDL	serum	CDV, CPV	Justice-Allen, A. and Clement, M.J., 2019. Effect of Canine Parvovirus and Canine Distemper Virus on the Mexican Wolf (Canis lupus baileyi) Population in the USA. Journal of Wildlife Diseases, 55(3), pp.682-688.
MI	Cornell	serum	CAV, CDV, CHV, CPV, NEO, TOXO (MAT)	This paper.
MT	Cornell	serum	CAV, CDV, CHV, CPV, NEO	This paper.
N NWT	Cornell	strips	CAV, CDV, CPV, NEO	This paper.
ONT	Cornell	serum	CAV, CDV, CHV, CPV, NEO, TOXO (MAT)	This paper.
SE AK	Cornell	serum	CAV, CDV, CHV, CPV, NEO, TOXO (MAT)	This paper.
SNF	CSU, Cornell	serum	CAV, CDV, CHV, CPV, NEO, TOXO (ELISA/MAT)	Carstensen, M., Giudice, J.H., Hildebrand, E.C., Dubey, J.P., Erb, J., Stark, D., Hart, J., Barber-Meyer, S., Mech, L.D., Windels, S.K. and Edwards, A.J., 2017. A serosurvey of diseases of free-ranging gray wolves (canis lupus) in Minnesota, USA. Journal of Wildlife Diseases, 53(3), pp.459-471.
SS NWT	Cornell	serum	CAV, CDV, CHV, CPV, NEO, TOXO (MAT)	This paper.
YNP	Cornell	serum	CAV, CDV, CHV, CPV, NEO, TOXO (ELISA/MAT)	This paper.
YUCH	Cornell	serum	CAV, CDV, CHV, CPV, NEO, TOXO (MAT)	This paper.

Table S3. Labs where samples were analyzed, the type of assay performed for each pathogen (CAV adenovirus, CDV distemper, CPV parvovirus, CHV herpesvirus, NEO *N. caninum*, TOXO *T. gondii*), the titer value in which a titer equal or above was considered seropositive, and the lab name and location. Assay types included VN virus/serum neutralization, HI hemagglutination inhibition, IFA indirect fluorescent assay, ELISA enzyme-linked immunosorbent assay, and MAT monocyte activation test. TOXO ELISA assays provide a negative/positive result, whereas TOXO MAT or IFA provide a titer result.

lab	CAV assay	CAV titer	CDV assay	CDV titer	CHV assay	CHV titer	CPV assay	CPV titer	NEO assay	NEO titer	TOXO assay	TOXO titer	lab name & location
Cornell: serum	VN	1:16	VN	1:16	VN	1:16	HI	1:20	IFA	NA	ELISA / MAT	1:25	Cornell Animal Health Diagnostic Center, Ithaca, New York, USA
Cornell: strips	VN	1:20	VN	1:20	VN	1:16	HI	1:20	IFA	NA	MAT	1:25	Cornell Animal Health Diagnostic Center, Ithaca, New York, USA
CSU	NA	NA	VN	1:25	NA	NA	HI	1:256	NA	NA	MAT	1:50	Colorado State University Veterinary Diagnostic Laboratory, Fort Collins, Colorado, USA
WCVM	NA	NA	VN	1:12	NA	NA	HI	1:40	NA	NA	NA	NA	Western College of Veterinary Medicine, Saskatoon, Saskatchewan, Canada
WADDL	VN	1:4	IFA	1:50	VN	1:4	IFA	1:25	IFA	1:50	IFA	1:64	Washington Animal Disease Diagnostic Laboratory, Pullman, Washington, USA
NMVDL (pre-July 2012)	NA	NA	VN / IFA	1:20	NA	NA	HI	1:80	NA	NA	NA	NA	New Mexico Veterinary Diagnostic Laboratory, Albuquerque, New Mexico, USA
NMVDL (post-July 2012)	NA	NA	ELISA	1:16	NA	NA	HI	1:80	NA	NA	NA	NA	New Mexico Veterinary Diagnostic Laboratory, Albuquerque, New Mexico, USA

Table S4. A data summary for each study area: total sample size from each study area, number of years sampled, and the proportion of missing data from the variables of interest (Table 2). Some variables were calculated for all study areas, thus were never missing (i.e., habitat quality, human density, prey species, latitude, longitude). Pack density, pack size, and wolf density were considered as annual estimates, so proportion missing is the number of years without estimates out of all years wolves were sampled; in the final models, we used average pack size and wolf density so there was no missingness for pack size and wolf density.

Study Area	Sample size	# years	Pack membership	Pack density	Pack size	Wolf density	Sex	Coat color	Age (numeric)	Age (category)	Social status
AK.PEN	100	6	0.05	1.00	0.00	0.00	0.00	0.00	0.01	0.01	0.59
BAN JAS	96	17	0.02	1.00	0.70	0.00	0.08	0.43	1.00	0.07	0.70
BC	145	11	0.01	1.00	0.20	0.40	0.00	0.01	0.70	0.14	0.85
DENALI	154	16	0.01	0.00	0.00	0.00	0.00	0.00	0.00	0.00	1.00
ELLESMERE	11	5	0.00	1.00	0.20	0.00	0.00	0.00	0.45	0.00	0.18
GTNP	60	7	0.00	0.00	0.29	0.00	0.00	0.00	0.00	0.00	0.32
INT AK	35	2	0.00	1.00	0.00	0.00	0.00	0.00	0.86	0.00	1.00
MEXICAN	181	14	0.01	1.00	0.00	0.00	0.00	0.00	0.01	0.01	1.00
MI	102	9	0.25	0.20	0.00	0.25	0.02	0.96	0.73	0.01	1.00
MT	351	15	1.00	1.00	0.00	0.00	0.03	1.00	0.26	0.13	1.00
N NWT	67	4	1.00	0.00	0.00	0.00	0.00	0.88	1.00	0.22	1.00
ONT	60	5	0.03	1.00	0.20	0.40	0.00	0.55	1.00	0.08	0.77
SE AK	10	4	0.00	1.00	0.67	0.33	0.00	0.00	0.14	0.14	1.00
SNF	92	8	0.79	0.00	0.00	0.00	0.00	0.35	0.02	0.00	1.00
SS NWT	34	3	0.05	1.00	0.00	0.25	0.00	0.00	0.19	0.19	0.00
YNP	383	25	0.00	0.00	0.00	0.00	0.00	0.00	0.00	0.00	0.13
YUCH	105	22	0.03	1.00	0.14	0.00	0.01	0.01	0.07	0.01	1.00

Table S5. Information about how most samples from each study area were collected and the corresponding ethics/approval.

Study Area	Live/Dead wolf	Permit type and number	Other approval / protocol followed
AK.PEN	Live		Alaska Department of Fish & Game Division of Wildlife Conservation Animal Care and Use Committee protocol # 06-19
BAN JAS	Live	University of Alberta 353112, Parks Canada Banff National Park 004753 and Jasper National Park 2007-952, Alberta GP4816 and CN 087,	

		WILKA101-07, British Columbia<br>VI07-31411	
BC	Live	BC 2(h) permit PG17-272811	
DENALI	Live	National Park Service Institutional Animal Care and Use Committee 2010-1, AKR_YUCH and DENA_Burch_Wolves_2013, AKR_DENA_Borg_Wolves_2016.A3, AKR_DENA_Borg_Wolves_2019.A3<br><br>Annual state of Alaska permits	
ELLESMERE	Live	Nunavut Wildlife Research Permits 2014-010, 2015-048, 2016-043, 2017-042, and 2018-036	
GTNP	Live	National Park Service Institutional Animal Care and Use Committee approval	
INT.AK	Live	Alaska Fish and Game Division of Wildlife Conservation Institutional Animal Care and Use Committee protocol # 0062-2019-28	
MEXICAN	Live	Federal endangered species permit # TE091551-0	
MI	Live		Michigan Department of Natural Resources Wildlife Veterinarian
MT	Live		BIOMEDICAL PROTOCOL FOR FREE-RANGING GRAY WOLVES (*Canis lupus*) IN MONTANA: Capture, anesthesia, surgery, tagging, sampling and necropsy procedures
N NWT	Dead (harvest)	Wildlife Research Permit # WL500666	Northwest Territories Wildlife Care Committee
ONT	Live	Ontario Ministry of Natural Resources permits 10-218, 11-218, 75-05, 75-06, 75-07, and 75-08	All animal capture and handling was in accordance with federal, provincial, and institutional animal care and use protocols
SE AK	Live		Alaska Fish and Game Animal Care and Use Committee protocol #2012–028, #2014–15, #0043-2017-43
SNF	Live	U.S. Fish and Wildlife Service permit TE3886A-0<br><br>Institutional Animal Care and Use Committee. 2015. Northern Prairie Wildlife Research Center, US Geological Survey, Jamestown, North Dakota, USA	
SS NWT	Live	Wildlife Research Permit # WL500666	Northwest Territories Wildlife Care Committee
YNP	Live	Institutional Animal Care and Use Committee permit IMR_YELL_Smith_wolves_2012	National Park Service veterinarian, Yellowstone National Park review committee and the Superintendent
YUCH	Live	National Park Service Institutional Animal Care and Use Committee AKR_YUCH.DENA_Burch _Wolves_2013.A3	

Titer cutoffs

We assessed the effect of titer cutoff on population pathogen seroprevalence. We used the serum datasets we tested at Cornell (Table S2) that titer data was provided (i.e., removed *N. caninum*, Table S3). Conservative cutoffs were one dilution above the standard cutoff. In general, seroprevalence was insensitive to titer cutoff; *T. gondii* was slightly sensitive to titer cutoff, but mean difference was still <6% (Fig. S1).

Titer thresholds defining positive/negative were as follows:

Standard CAV, CDV, CHV ≥1:16

Standard CPV ≥1:20

Standard *T. gondii* ≥1:25

Conservative CAV, CDV, CHV ≥1:24

Conservative CPV ≥1:40

Conservative *T. gondii* ≥1:50

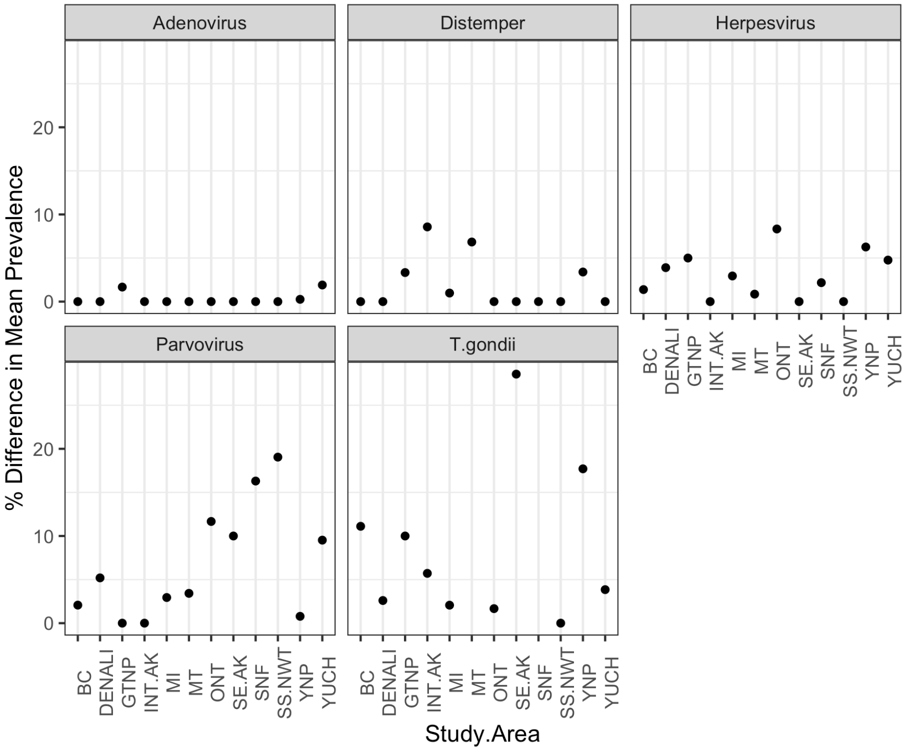

Figure S1. Percent difference in mean pathogen seroprevalence using the standard and conservative titer cutoffs by study area.

Figure S2 (next page). Annual pathogen seroprevalence by study area: (A) adenovirus, (B) distemper, (C) herpesvirus, (D) parvovirus, (E) *N. caninum* and (F) *T. gondii*. Note that not all populations were sampled over the entire span of years displayed on the x-axis.

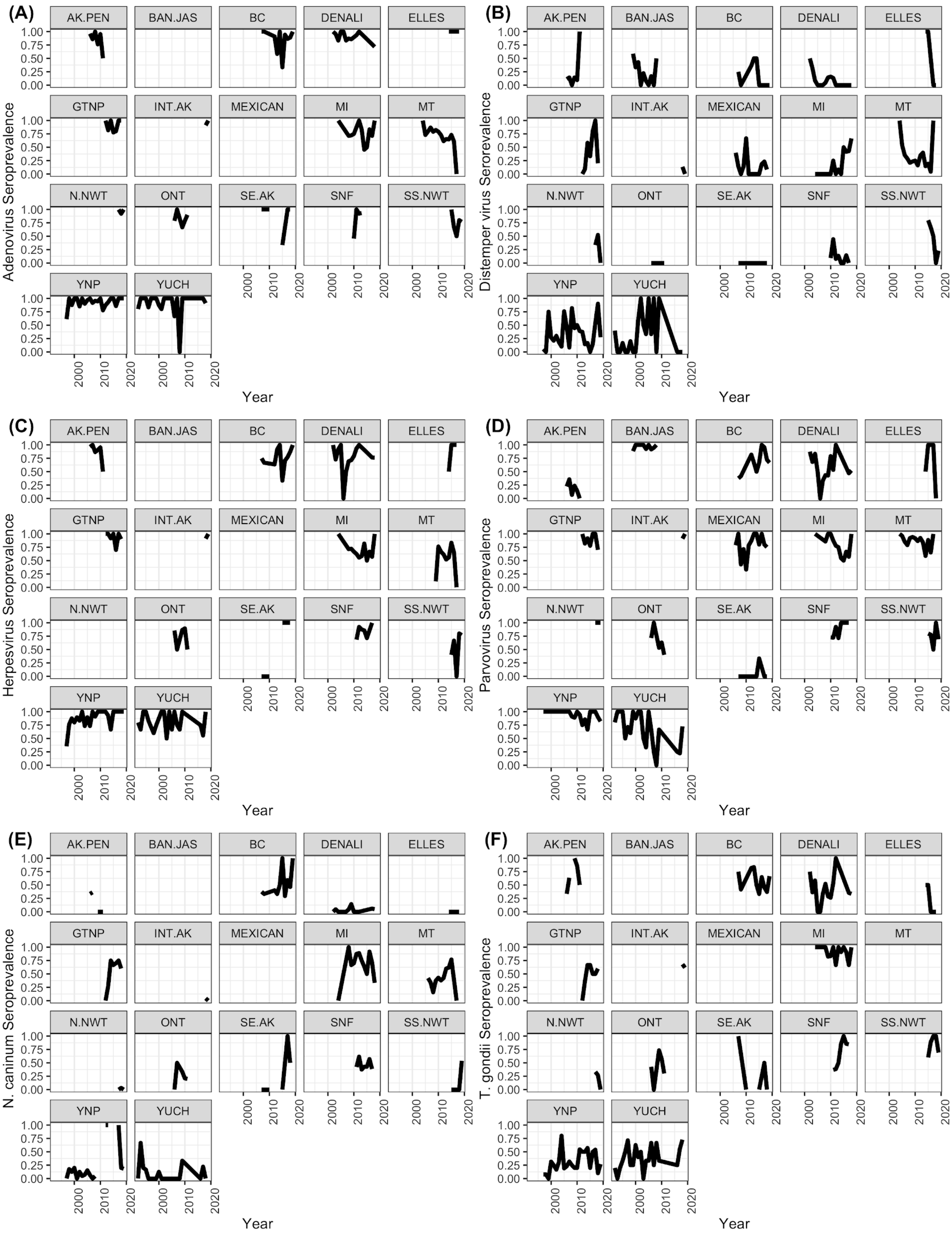

(A)
AK.PEN
BAN.JAS
BC
DENALI
ELLES
GTNP
INT.AK
MEXICAN
MI
MT
N.NWT
ONT
SE.AK
SNF
SS.NWT
YNP
YUCH
Adenovirus Seroprevalence
Year
(B)
Distemper virus Serorevalence
Year
(C)
Herpesvirus Seroprevalence
Year
(D)
Parvovirus Seroprevalence
Year
(E)
N. caninum Seroprevalence
Year
(F)
T. gondii Seroprevalence
Year
0.00
0.25
0.50
0.75
1.00
2000
2010
2020

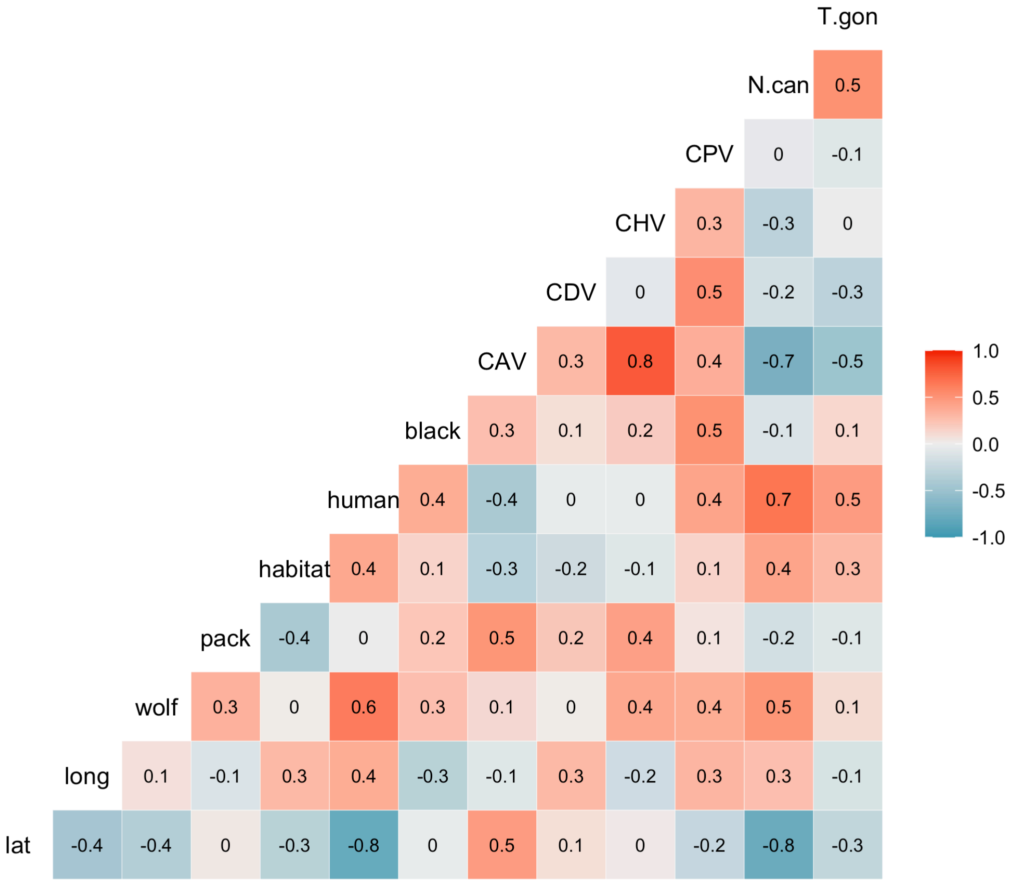

Figure S3. Correlation matrix (Spearman's) for standardized continuous predictor variables and mean pathogen seroprevalence: latitude (lat), longitude (long), wolf density (wolf), pack size (pack), habitat quality (habitat), human density (human), black coat color (i.e., proportion black) (black), canine adenovirus (CAV), canine distemper virus (CDV), canine herpesvirus (CHV), canine parvovirus (CPV), *N. caninum* (N.can), and *T. gondii* (T.gon).

Model evaluation

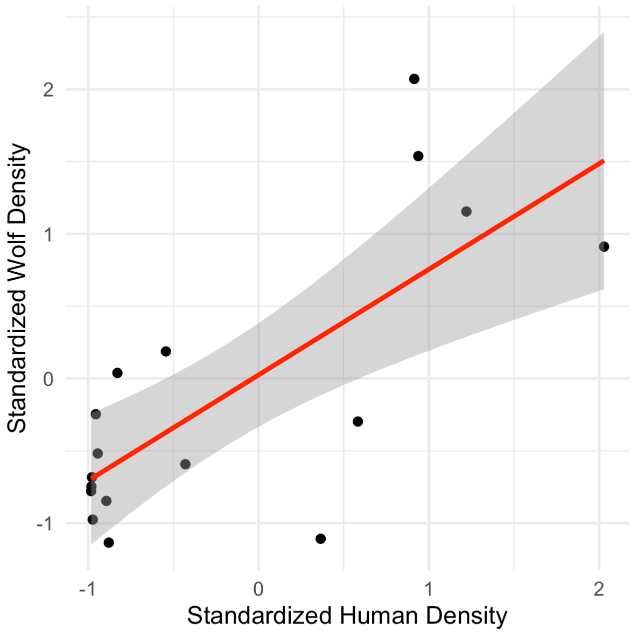

Figure S4. A plot of the relationship between standardized human density and standardized wolf density. Each point represents one study area. The red line is the fitted line from a simple linear regression with a 95% confidence interval: $R^2 = 0.54$, $p < 0.001$.

4-fold cross validation groups

We retained geographical diversity within both the training and the testing sets. Because we have 17 study areas and a 4-fold validation, one population (BC) is not in the testing set.

GROUP 1
Train: AK.PEN, DENALI, INT.AK, ELLESMERE, SS.NWT, BC, SE.AK, BAN.JAS, YNP, GTNP, MEXICAN, SNF, MI
Test: YUCH, N.NWT, MT, ONT

GROUP 2
Train: AK.PEN, YUCH, INT.AK, N.NWT, SS.NWT, BC, SE.AK, BAN.JAS, MT, GTNP, MEXICAN, ONT, MI
Test: DENALI, ELLESMERE, YNP, SNF

GROUP 3
Train: DENALI, YUCH, INT.AK, ELLESMERE, N.NWT, SS.NWT, BC, BAN.JAS, YNP, MT, MEXICAN, SNF, ONT
Test: AK.PEN, SE.AK, GTNP, MI

GROUP 4
Train: AK.PEN, DENALI, YUCH, ELLESMERE, N.NWT, BC, MT, YNP, GTNP, SE.AK, SNF, MI, ONT
Test: INT.AK, SS.NWT, BAN.JAS, MEXICAN

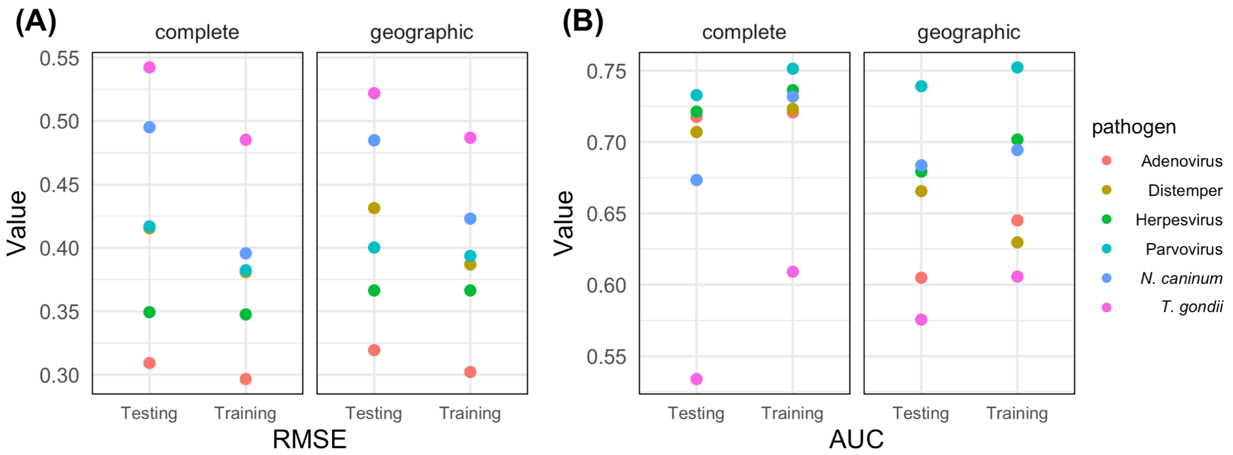

Figure S5. Model evaluation criteria using training and testing sets: (A) root mean square error (RMSE) and (B) area under the receiver-operator curve (AUC) for each pathogen (colored dots) and model (panels: *complete* and *geographic*). Each estimate is the mean of the four estimates from the four-fold cross validation procedure (Table S6).

Table S6. A list of model evaluation results from the *geographic* and *complete* models for each pathogen analyzed. Root mean squared error (RMSE) and area under the receiver-operator curve (AUC) were measured for both training and testing datasets – the average RMSE or AUC for the four training and testing sets is displayed (see training/testing sets above).

pathogen	model	RMSE (training set)	RMSE (testing set)	AUC (training set)	AUC (testing set)
adenovirus	geographic	0.3021	0.3194	0.645	0.6049
adenovirus	complete	0.2965	0.3092	0.7208	0.7176
distemper virus	geographic	0.3869	0.4313	0.6296	0.6656
distemper virus	complete	0.3808	0.4154	0.7232	0.707
herpesvirus	geographic	0.3664	0.3664	0.7017	0.6793
herpesvirus	complete	0.3475	0.3493	0.7363	0.7212
parvovirus	geographic	0.4230	0.4848	0.6944	0.6837
parvovirus	complete	0.3956	0.4951	0.7318	0.6734
N. caninum	geographic	0.3936	0.4002	0.7522	0.7390
N. caninum	complete	0.3823	0.4168	0.7513	0.7328
T. gondii	geographic	0.4868	0.5219	0.6057	0.5756
T. gondii	complete	0.4852	0.5422	0.6091	0.5339

Model Results

Table S7. A list of results for each variable in models predicting pathogen exposure. For each variable, the coefficient estimate (β), standard error (in parentheses), and p-value (p) are listed. Categorical variables are interpreted as the effect of: gray wolves with respect to black, males with respect to females, and pups and subadults with respect to adults.

variable	adenovirus	distemper	herpesvirus	parvovirus	*N. caninum*	*T. gondii*
**Latitude**	β=-0.03 (0.12) p=0.78	β=0.03 (0.24) p=0.91	β=-0.21 (0.13) p=0.10	β=-0.17 (0.18) p=0.34	β=-1.08 (0.33) p=<0.01	β=-0.22 (0.15) p=0.16
**Longitude**	β=-0.07 (0.09) p=0.42	β=0.17 (0.25) p=0.50	β=-0.21 (0.10) p=0.03	β=0.25 (0.17) p=0.15	β=0.07 (0.24) p=0.78	β=-0.05 (0.12) p=0.66
**Habitat quality**	β=-0.09 (0.10) p=0.36	β=-0.52 (0.27) p=0.05	β=-0.12 (0.11) p=0.29	β=0.06 (0.24) p=0.81	β=0.16 (0.37) p=0.64	β=-0.08 (0.14) p=0.58
**Human density**	β=0.25 (0.10) p=0.01	β=0.85 (0.21) p=<0.01	β=0.21 (0.10) p=0.04	β=0.52 (0.27) p=0.05	β=0.98 (0.40) p=0.01	β=0.02 (0.15) p=0.91
**Wolf density**	β=0.24 (0.09) p=0.01	β=0.52 (0.27) p=0.05	β=0.25 (0.10) p=0.01	β=0.38 (0.28) p=0.17	β=0.87 (0.36) p=0.02	β=-0.02 (0.14) p=0.92
**Pack size**	β=0.15 (0.15) p=0.31	β=-0.04 (0.28) p=0.89	β=0.25 (0.16) p=0.12	β=0.04 (0.28) p=0.89	β=-0.25 (0.50) p=0.62	β=-0.13 (0.20) p=0.50
**Color - grey**	β=0.17 (0.10) p=0.10	β=-0.17 (0.16) p=0.29	β=-0.09 (0.10) p=0.36	β=-0.01 (0.12) p=0.93	β=0.38 (0.19) p=0.05	β=-0.10 (0.12) p=0.38
**Sex - male**	β=-0.09 (0.09) p=0.35	β=0.06 (0.14) p=0.69	β=-0.07 (0.09) p=0.45	β=0.02 (0.10) p=0.81	β=0.03 (0.16) p=0.83	β=-0.12 (0.11) p=0.26
**Pups**	β=-0.95 (0.15) p=<0.01	β=-2.22 (0.23) p=<0.01	β=-1.09 (0.14) p=<0.01	β=-1.53 (0.18) p=<0.01	β=-0.75 (0.27) p=<0.01	β=-0.42 (0.16) p=0.01
**Subadults**	β=-0.34 (0.12) p=<0.01	β=-1.05 (0.17) p=<0.01	β=-0.67 (0.12) p=<0.01	β=-0.67 (0.14) p=<0.01	β=-0.48 (0.21) p=0.02	β=-0.32 (0.13) p=0.01

Canine adenovirus

Geographic model pseudo-R^2 (Cragg-Uhler): 0.12

Complete model pseudo-R^2 (Cragg-Uhler): 0.13

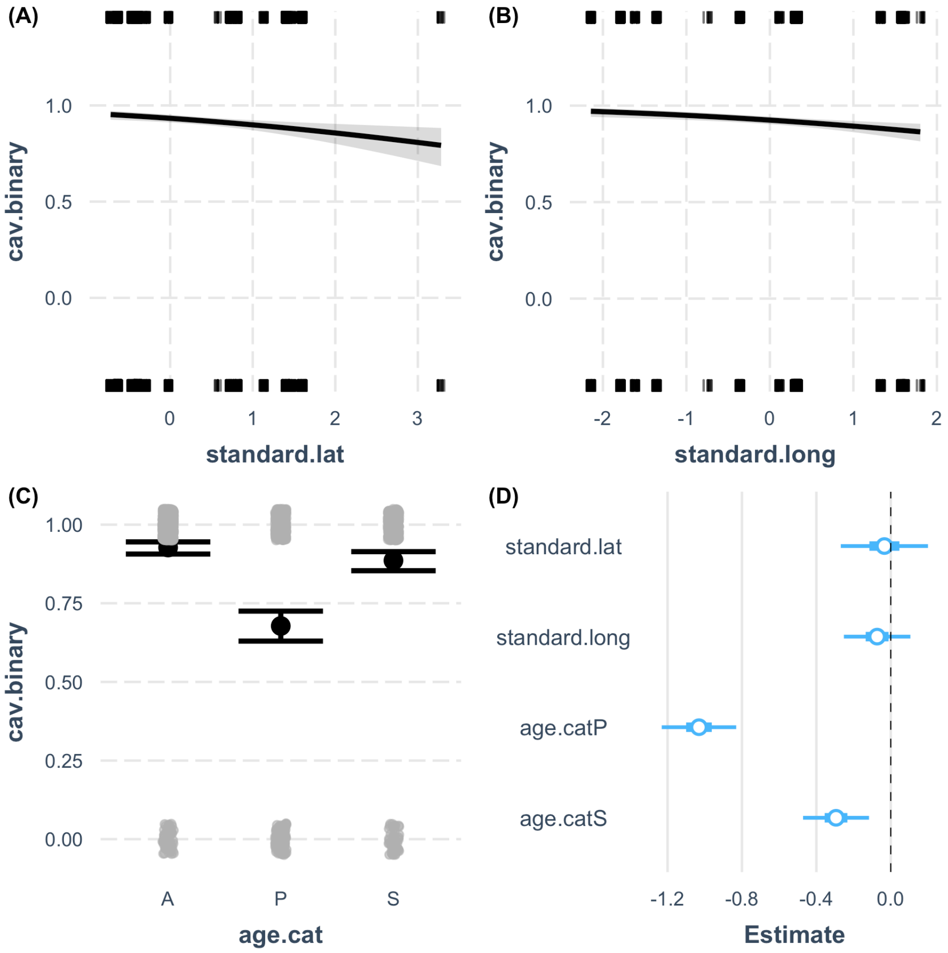

Figure S6. GLMM results from the *geographic model* predicting canine adenovirus exposure (cav.binary) with the predictor variables: (A) standardized latitude, (B) standardized longitude, and (C) age category (A=adult, P=pup, S=subadult), with 95% confidence intervals. Data are shown as a (A,B) rug plot or (C) gray points. (D) Coefficient estimates (circles) with 50% (thick lines) and 95% (thin lines) confidence intervals. If the intervals overlap with the vertical dashed line at zero, the variable is not considered statistically significant.

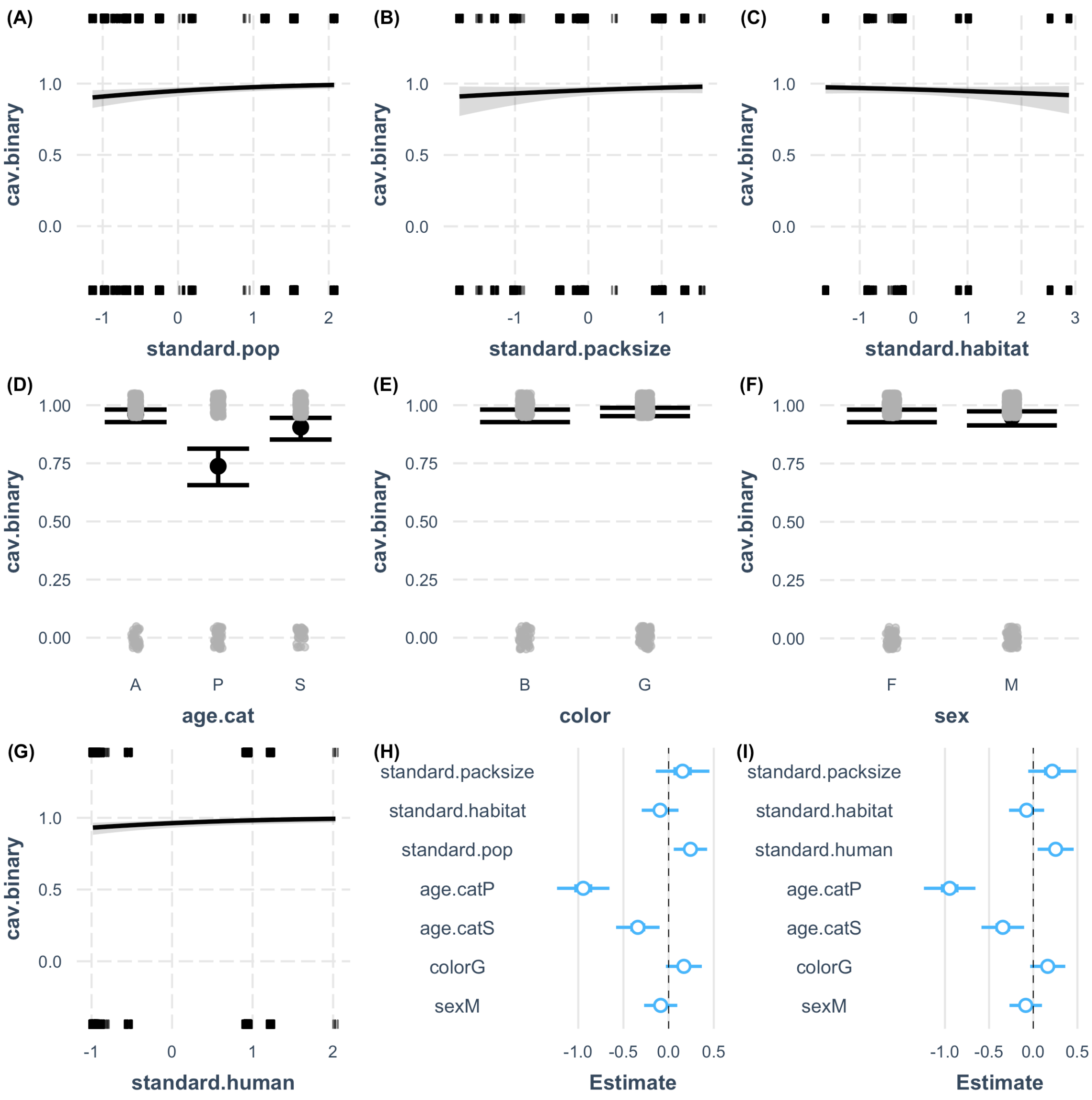

Figure S7. GLMM results from the *complete model* predicting canine adenovirus exposure (cav.binary) with the predictor variables: (A) wolf population density, (B) mean pack size, (C) habitat quality, (D) age category (A=adult, P=pup, S=subadult), (E) color (B=black, G=gray), (F) sex (F=female, M=male), and (G) human density, with 95% confidence intervals. Data are shown as a (A,B,C,G) rug plot or (D,E,F) gray points. Coefficient estimates (circles) with 50% (thick lines) and 95% (thin lines) confidence intervals for the *complete model* are shown including (H) wolf density and (I) human density. If the intervals overlap with the vertical dashed line at zero, the variable is not considered statistically significant. Categorical variables can be interpreted as: Pup and Subadult compared to Adults, Gray compared to Black, and Males compared to Females.

Canine distemper virus

Geographic model pseudo-R^2 (Cragg-Uhler): 0.09

Complete model pseudo-R^2 (Cragg-Uhler): 0.19

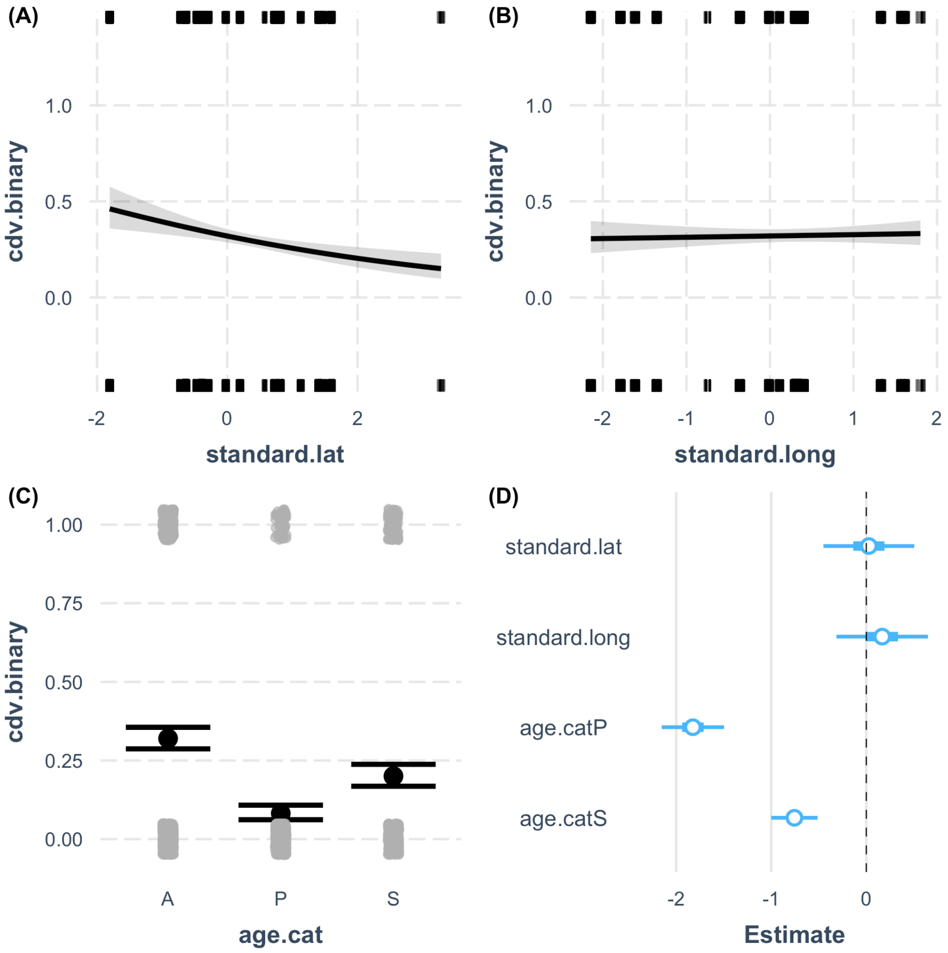

Figure S8. GLMM results from the *geographic model* predicting canine distemper virus exposure (cdv.binary) with the predictor variables: (A) standardized latitude, (B) standardized longitude, and (C) age category (A=adult, P=pup, S=subadult), with 95% confidence intervals. Data are shown as a (A,B) rug plot or (C) gray points. (D) Coefficient estimates (circles) with 50% (thick lines) and 95% (thin lines) confidence intervals. If the intervals overlap with the vertical dashed line at zero, the variable is not considered statistically significant.

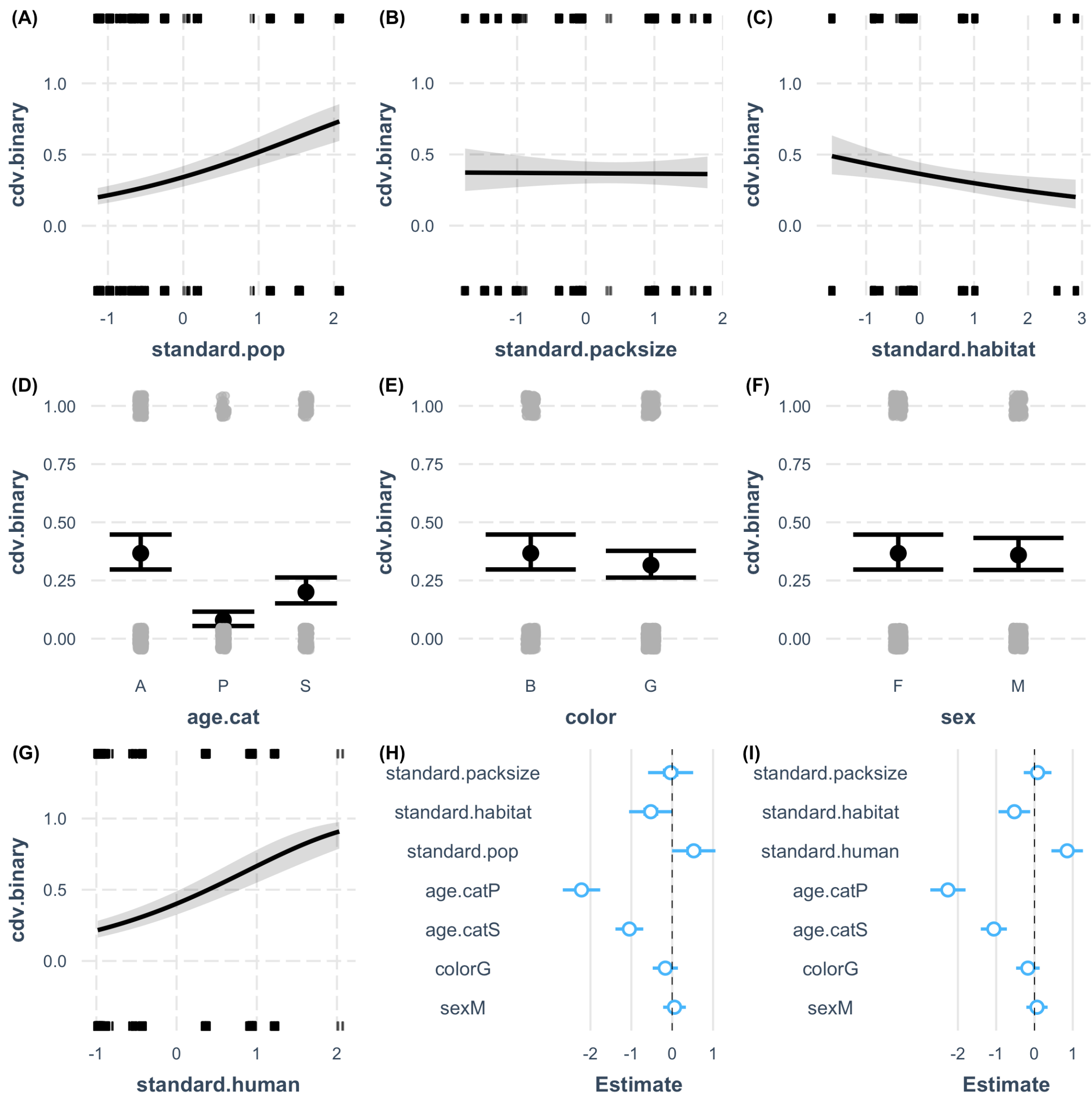

Figure S9. GLMM results from the *complete model* predicting canine dismpter virus exposure (cdv.binary) with the predictor variables: (A) wolf population density, (B) mean pack size, (C) habitat quality, (D) age category (A=adult, P=pup, S=subadult), (E) color (B=black, G=gray), (F) sex (F=female, M=male), and (G) human density, with 95% confidence intervals. Data are shown as a (A,B,C,G) rug plot or (D,E,F) gray points. Coefficient estimates (circles) with 50% (thick lines) and 95% (thin lines) confidence intervals for the *complete model* are shown including (H) wolf density and (I) human density. If the intervals overlap with the vertical dashed line at zero, the variable is not considered statistically significant. Categorical variables can be interpreted as: Pup and Subadult compared to Adults, Gray compared to Black, and Males compared to Females.

Canine herpesvirus
Geographic model pseudo-R² (Cragg-Uhler): 0.13
Complete model pseudo-R² (Cragg-Uhler): 0.17

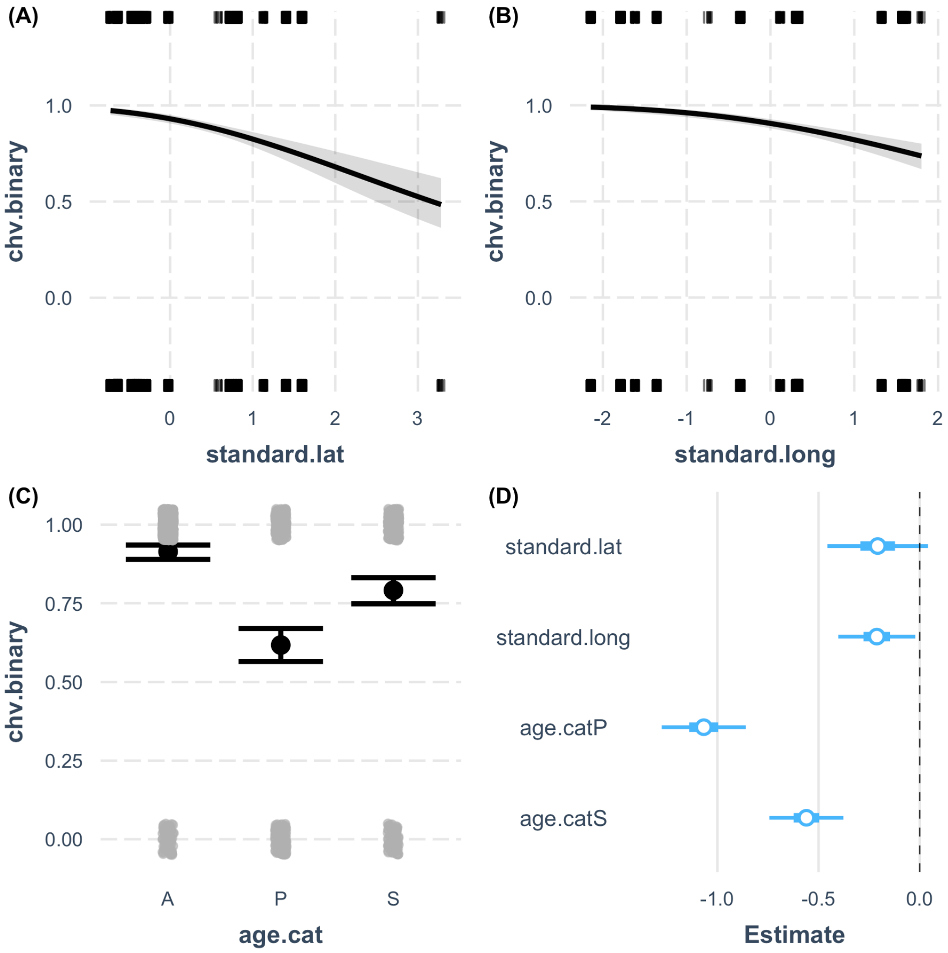

Figure S10. GLMM results from the *geographic model* predicting canine herpesvirus exposure (chv.binary) with the predictor variables: (A) standardized latitude, (B) standardized longitude, and (C) age category (A=adult, P=pup, S=subadult), with 95% confidence intervals. Data are shown as a (A,B) rug plot or (C) gray points. (D) Coefficient estimates (circles) with 50% (thick lines) and 95% (thin lines) confidence intervals. If the intervals overlap with the vertical dashed line at zero, the variable is not considered statistically significant.

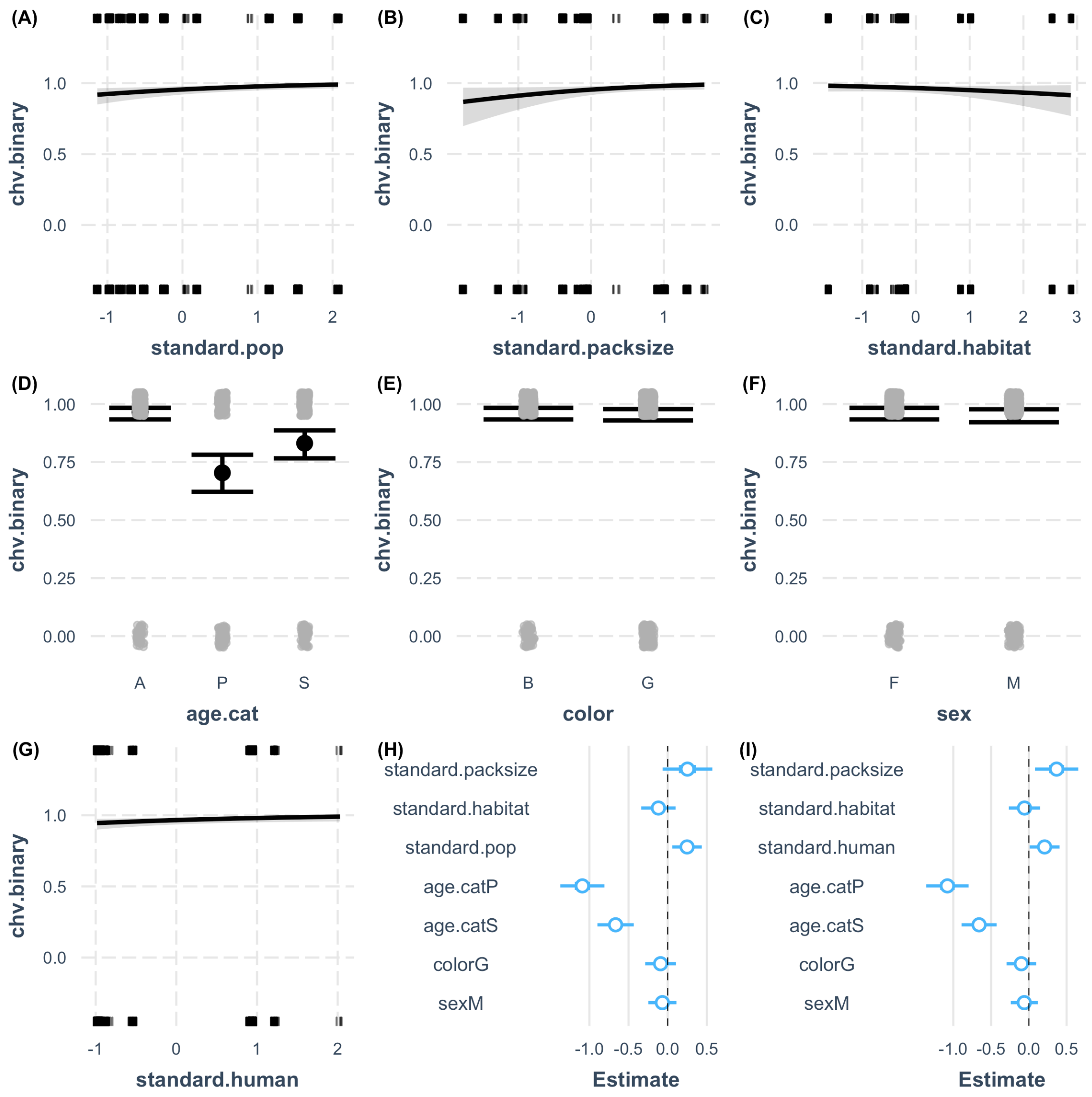

Figure S11. GLMM results from the *complete model* predicting canine herpesvirus exposure (chv.binary) with the predictor variables: (A) wolf population density, (B) mean pack size, (C) habitat quality, (D) age category (A=adult, P=pup, S=subadult), (E) color (B=black, G=gray), (F) sex (F=female, M=male), and (G) human density, with 95% confidence intervals. Data are shown as a (A,B,C,G) rug plot or (D,E,F) gray points. Coefficient estimates (circles) with 50% (thick lines) and 95% (thin lines) confidence intervals for the *complete model* are shown including (H) wolf density and (I) human density. If the intervals overlap with the vertical dashed line at zero, the variable is not considered statistically significant. Categorical variables can be interpreted as: Pup and Subadult compared to Adults, Gray compared to Black, and Males compared to Females.

Canine parvovirus

Geographic model pseudo-R^2 (Cragg-Uhler): 0.18

Complete model pseudo-R^2 (Cragg-Uhler): 0.18

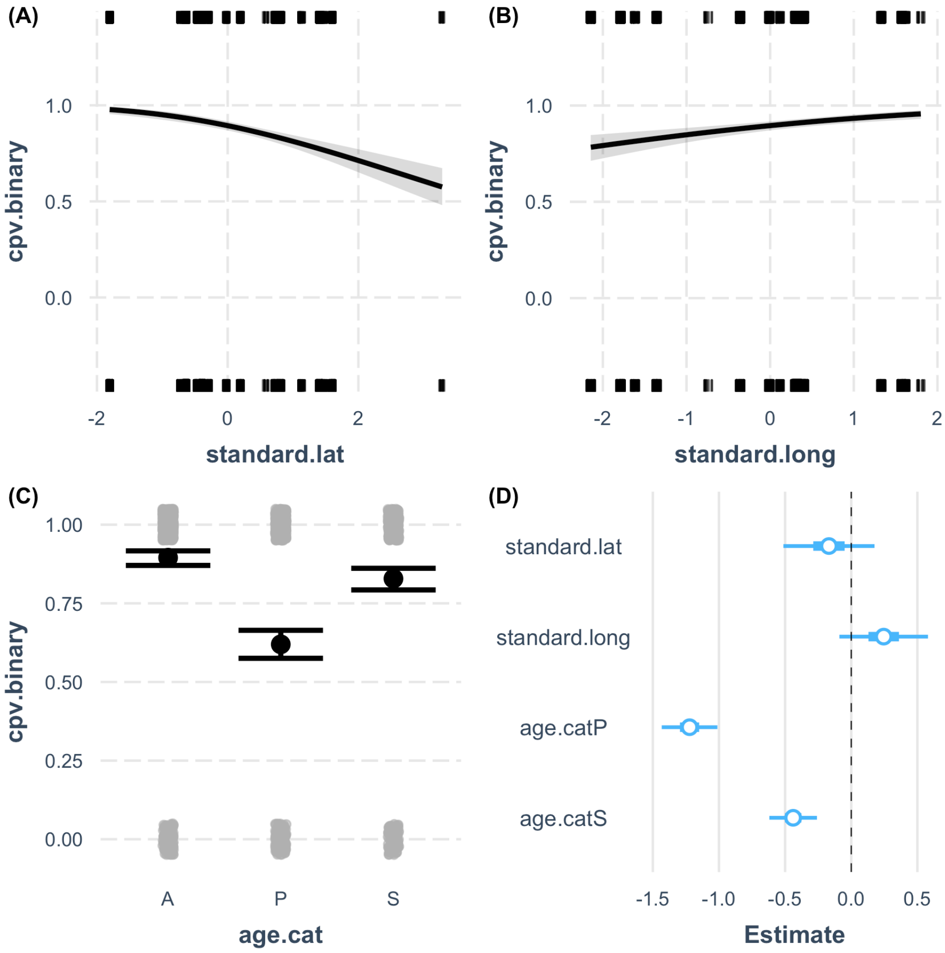

Figure S12. GLMM results from the *geographic model* predicting canine parvovirus exposure (cpv.binary) with the predictor variables: (A) standardized latitude, (B) standardized longitude, and (C) age category (A=adult, P=pup, S=subadult), with 95% confidence intervals. Data are shown as a (A,B) rug plot or (C) gray points. (D) Coefficient estimates (circles) with 50% (thick lines) and 95% (thin lines) confidence intervals. If the intervals overlap with the vertical dashed line at zero, the variable is not considered statistically significant.

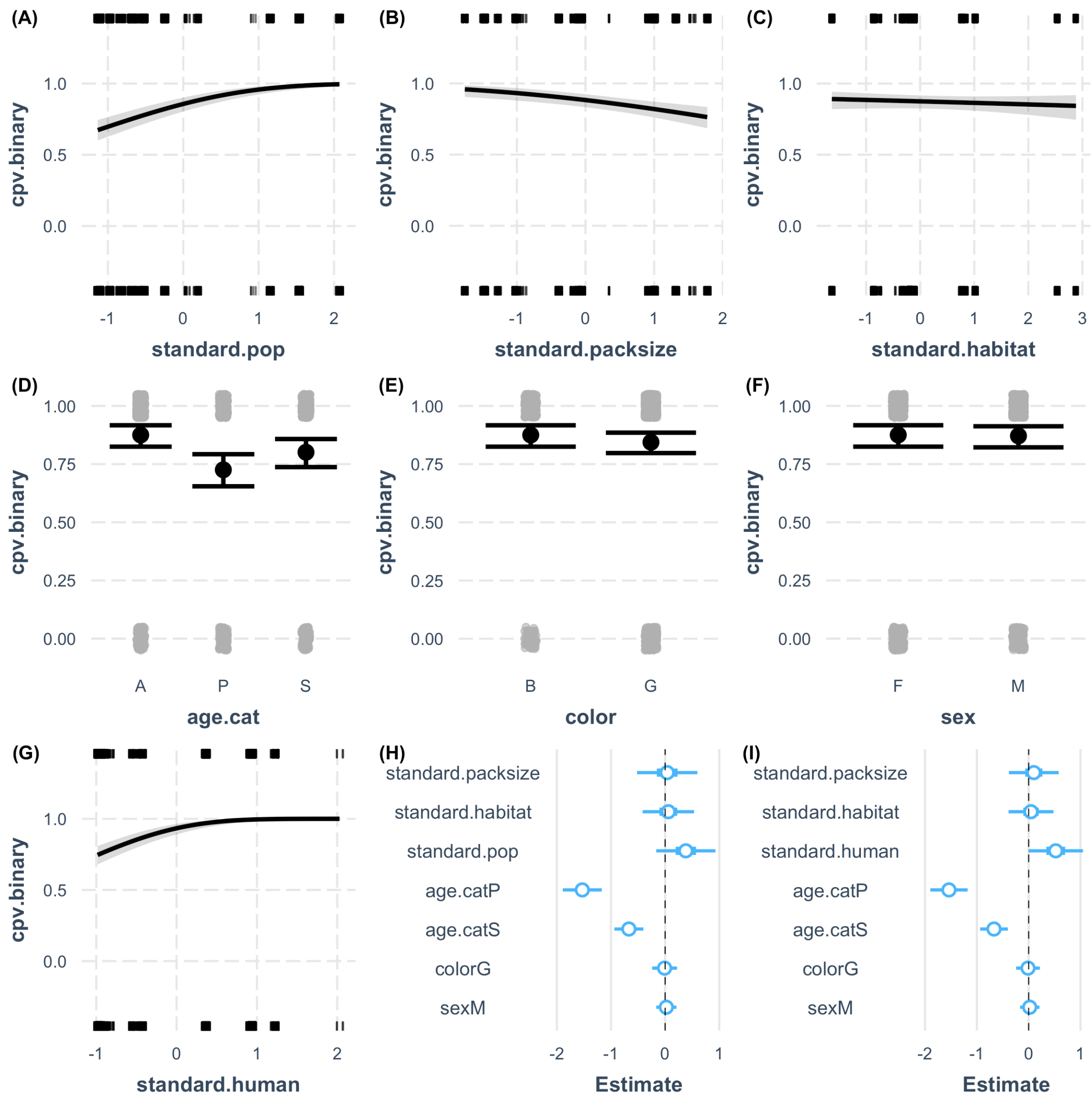

Figure S13. GLMM results from the *complete model* predicting canine parvovirus exposure (cpv.binary) with the predictor variables: (A) wolf population density, (B) mean pack size, (C) habitat quality, (D) age category (A=adult, P=pup, S=subadult), (E) color (B=black, G=gray), (F) sex (F=female, M=male), and (G) human density, with 95% confidence intervals. Data are shown as a (A,B,C,G) rug plot or (D,E,F) gray points. Coefficient estimates (circles) with 50% (thick lines) and 95% (thin lines) confidence intervals for the *complete model* are shown including (H) wolf density and (I) human density. If the intervals overlap with the vertical dashed line at zero, the variable is not considered statistically significant. Categorical variables can be interpreted as: Pup and Subadult compared to Adults, Gray compared to Black, and Males compared to Females.

Neospora caninum

Geographic model pseudo-R² (Cragg-Uhler): 0.19

Complete model pseudo-R² (Cragg-Uhler): 0.16

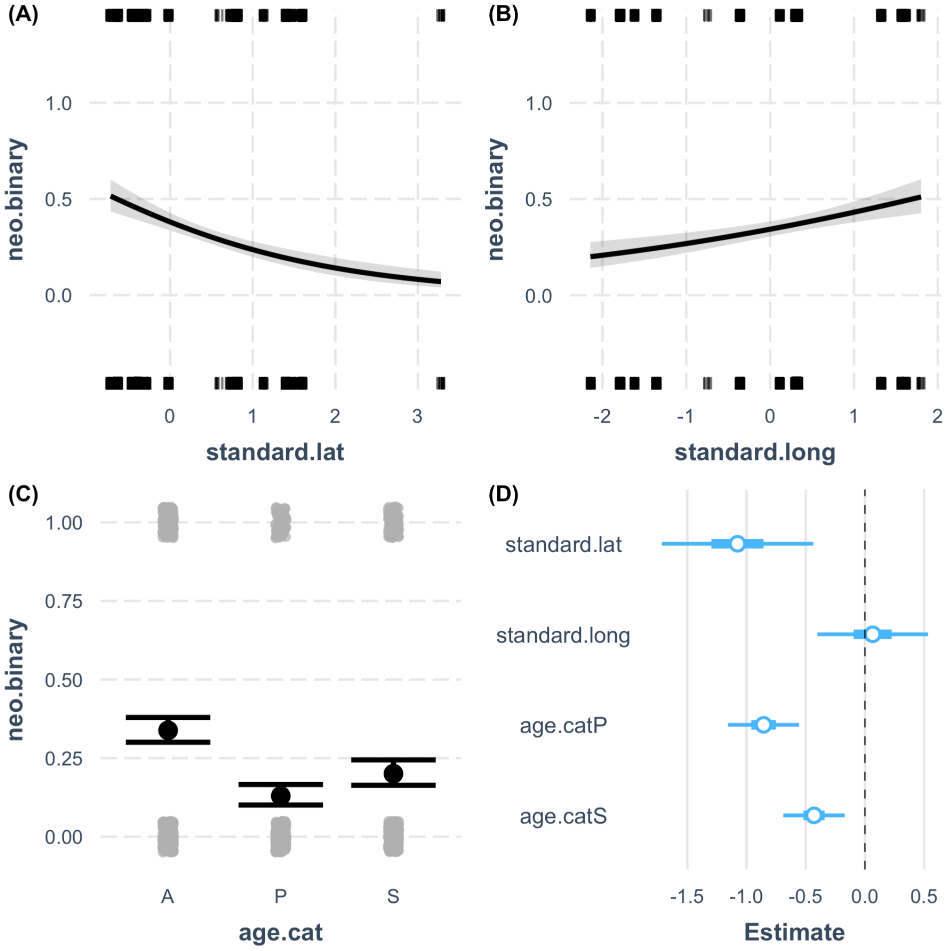

Figure S14. GLMM results from the *geographic model* predicting *N. caninum* exposure (neo.binary) with the predictor variables: (A) standardized latitude, (B) standardized longitude, and (C) age category (A=adult, P=pup, S=subadult), with 95% confidence intervals. Data are shown as a (A,B) rug plot or (C) gray points. (D) Coefficient estimates (circles) with 50% (thick lines) and 95% (thin lines) confidence intervals. If the intervals overlap with the vertical dashed line at zero, the variable is not considered statistically significant.

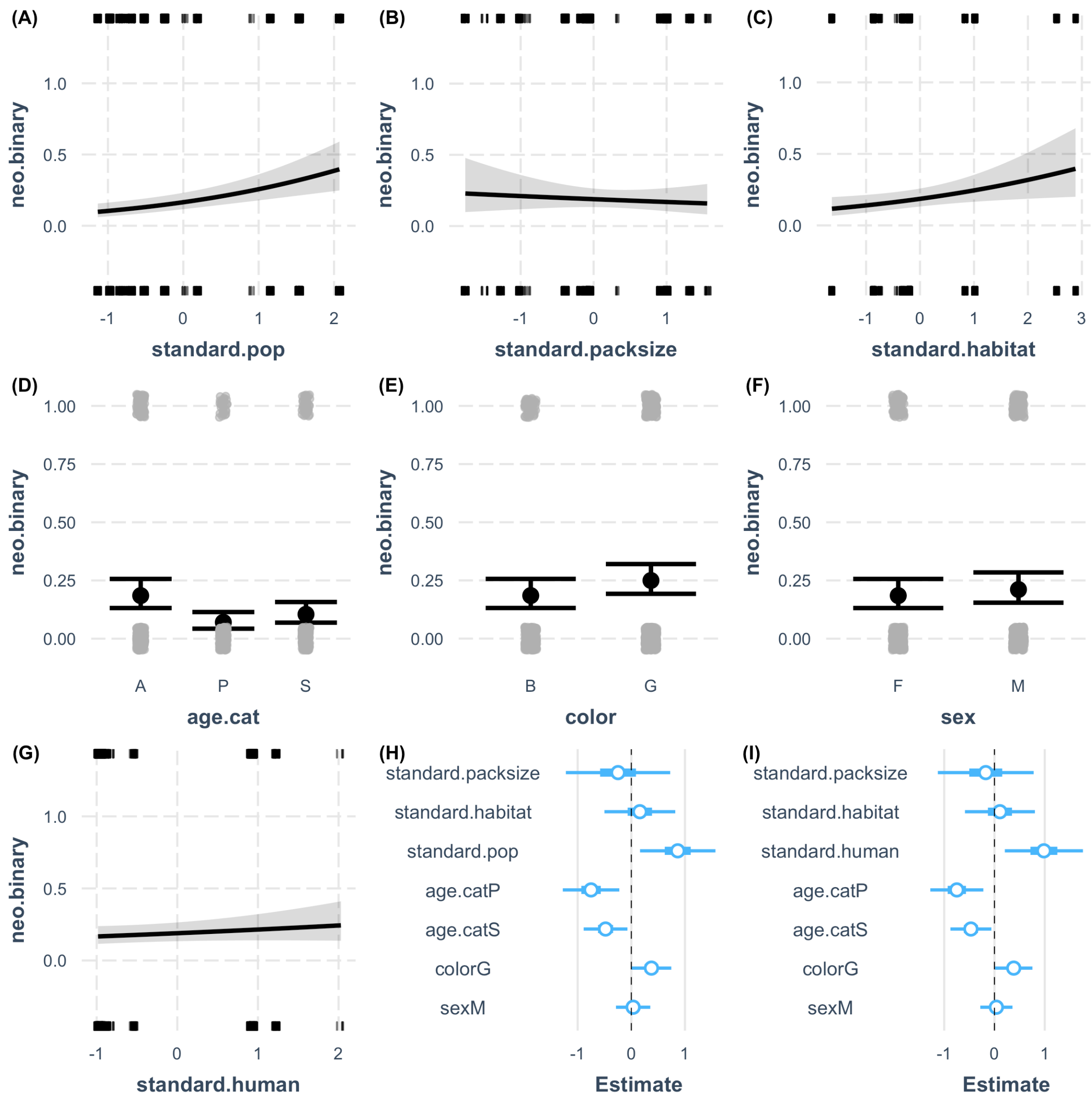

Figure S15. GLMM results from the *complete model* predicting *N. caninum* exposure (neo.binary) with the predictor variables: (A) wolf population density, (B) mean pack size, (C) habitat quality, (D) age category (A=adult, P=pup, S=subadult), (E) color (B=black, G=gray), (F) sex (F=female, M=male), and (G) human density, with 95% confidence intervals. Data are shown as a (A,B,C,G) rug plot or (D,E,F) gray points. Coefficient estimates (circles) with 50% (thick lines) and 95% (thin lines) confidence intervals for the *complete model* are shown including (H) wolf density and (I) human density. If the intervals overlap with the vertical dashed line at zero, the variable is not considered statistically significant. Categorical variables can be interpreted as: Pup and Subadult compared to Adults, Gray compared to Black, and Males compared to Females.

Toxoplasma gondii

Geographic model pseudo-R^2 (Cragg-Uhler): 0.04

Complete model pseudo-R^2 (Cragg-Uhler): 0.04

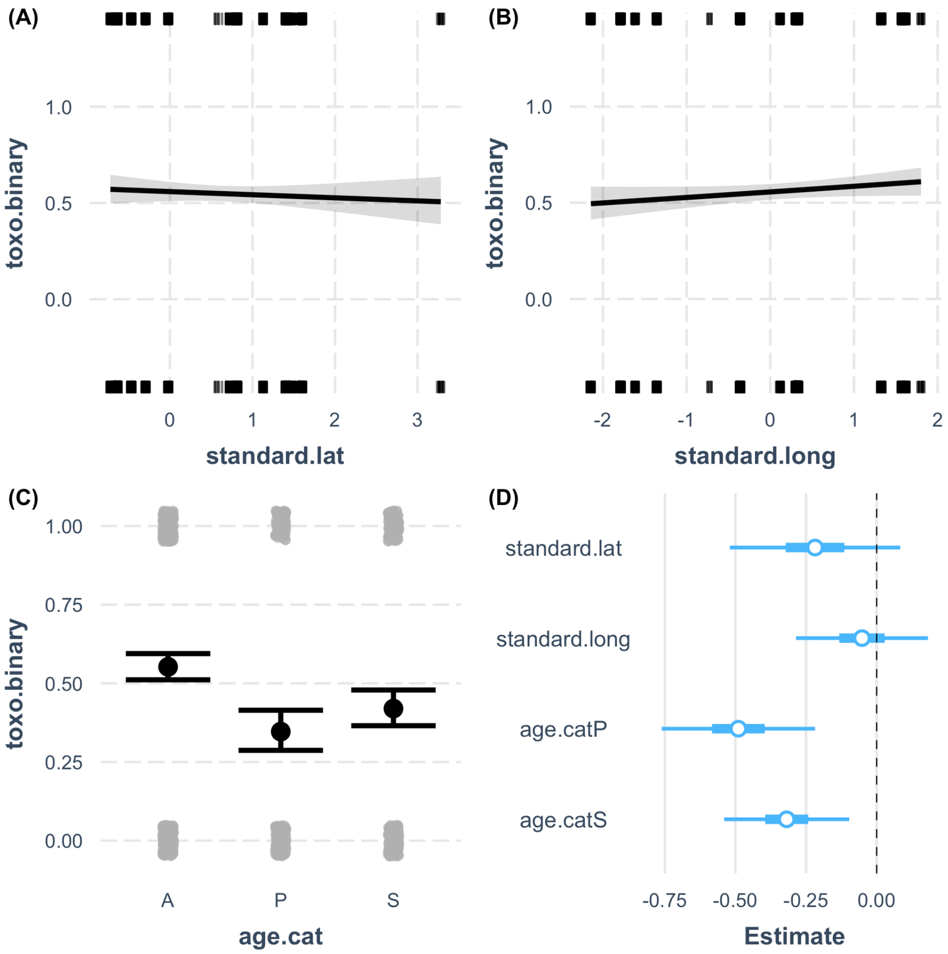

Figure S16. GLMM results from the *geographic model* predicting *T. gondii* exposure (toxo.binary) with the predictor variables: (A) standardized latitude, (B) standardized longitude, and (C) age category (A=adult, P=pup, S=subadult), with 95% confidence intervals. Data are shown as a (A,B) rug plot or (C) gray points. (D) Coefficient estimates (circles) with 50% (thick lines) and 95% (thin lines) confidence intervals. If the intervals overlap with the vertical dashed line at zero, the variable is not considered statistically significant.

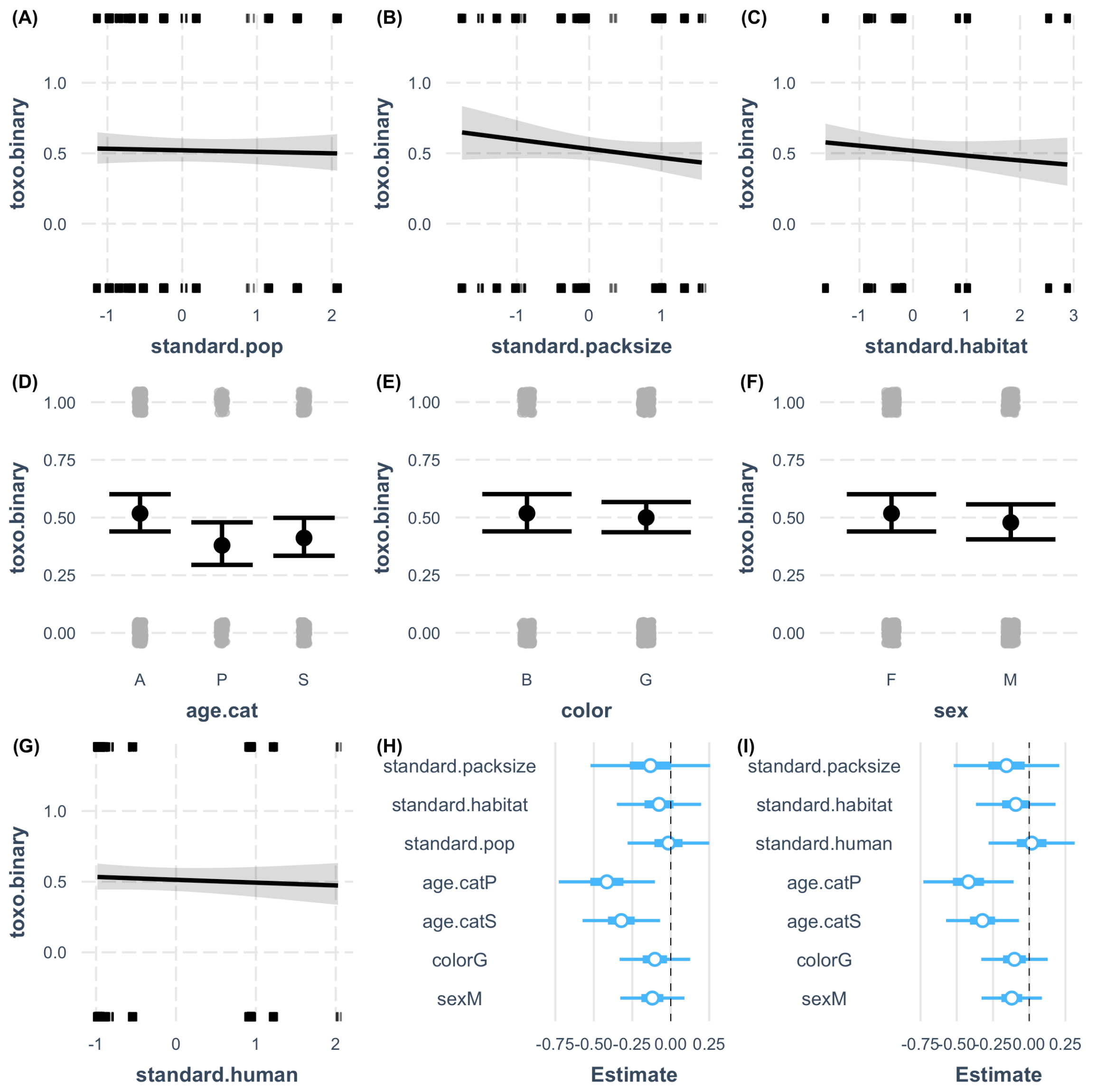

Figure S17. GLMM results from the *complete model* predicting *T. gondii* exposure (toxo.binary) with the predictor variables: (A) wolf population density, (B) mean pack size, (C) habitat quality, (D) age category (A=adult, P=pup, S=subadult), (E) color (B=black, G=gray), (F) sex (F=female, M=male), and (G) human density, with 95% confidence intervals. Data are shown as a (A,B,C,G) rug plot or (D,E,F) gray points. Coefficient estimates (circles) with 50% (thick lines) and 95% (thin lines) confidence intervals for the *complete model* are shown including (H) wolf density and (I) human density. If the intervals overlap with the vertical dashed line at zero, the variable is not considered statistically significant. Categorical variables can be interpreted as: Pup and Subadult compared to Adults, Gray compared to Black, and Males compared to Females.

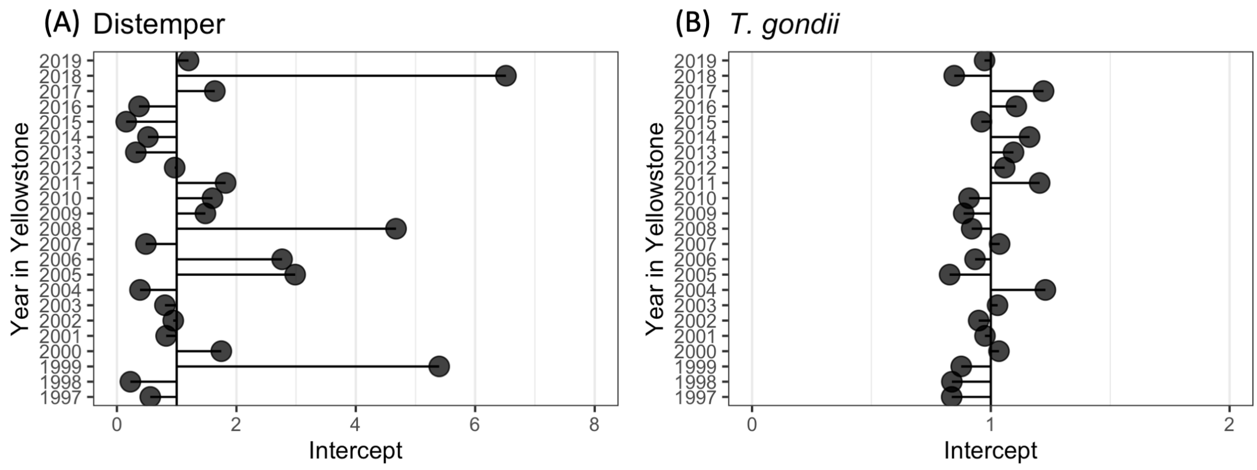

Figure S18. Plots displaying annual random intercepts for (A) canine distemper and (B) *T. gondii* in Yellowstone wolves, estimated using the *complete model*. Intercept estimates are in terms of odds (i.e., exponentiated) and points can be compared to the average year (vertical line at one).

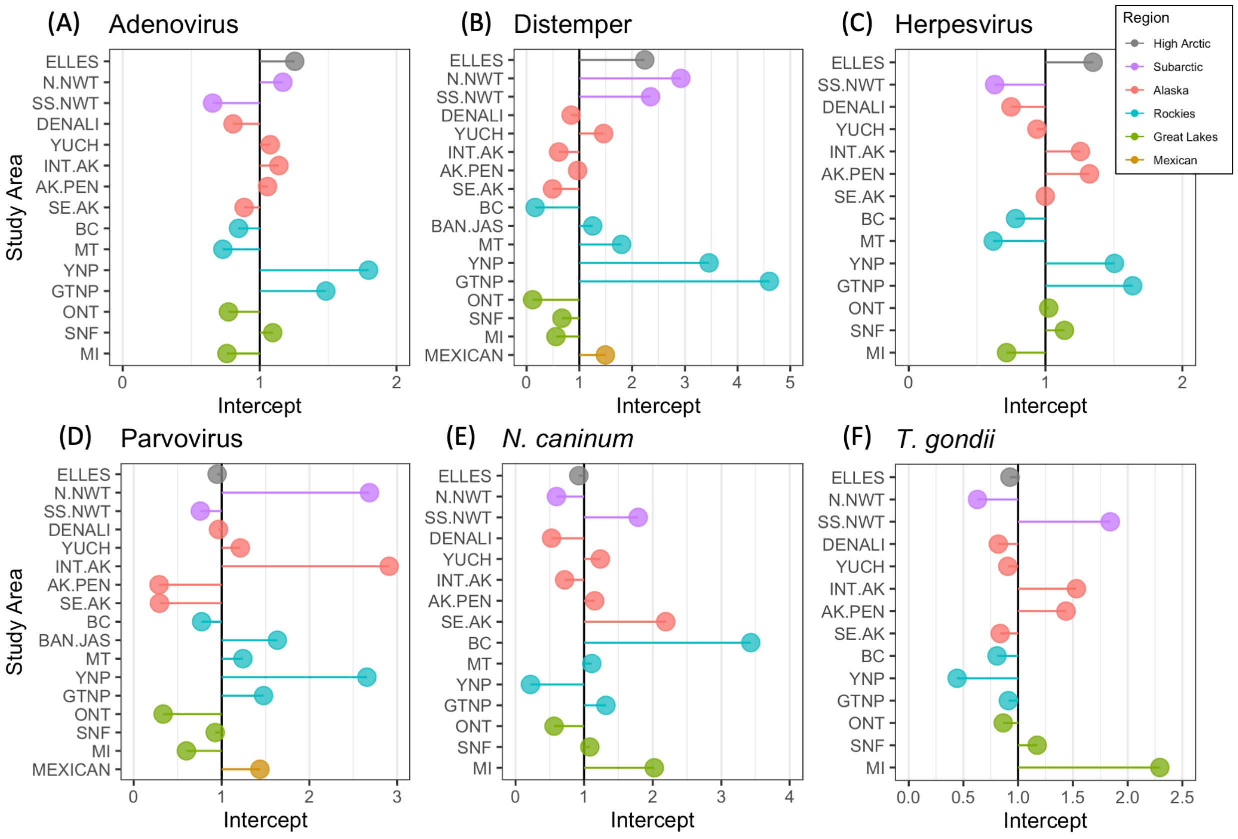

Figure S19. Plots of random intercept estimates for *geographic models* predicting exposure to (A) adenovirus, (B) distemper, (C) herpesvirus, (D) parvovirus, (E) *N. caninum*, and (F) *T. gondii* for each study area. Intercept estimates are in terms of odds (i.e., exponentiated) such that values less than one indicate that wolves in that study area have lower odds of exposure than the average of all wolves sampled (i.e., grand mean intercept), and values greater than one have

greater odds of exposure. Study areas are arranged from approximately north to south and grouped by region: High Arctic (gray), Subarctic (purple), Alaska (red), central Rocky Mountains (turquoise), Great Lakes (green), and Mexican (gold) (see Fig. 1 caption for study area abbreviations). Note that not all study areas were tested for each pathogen.

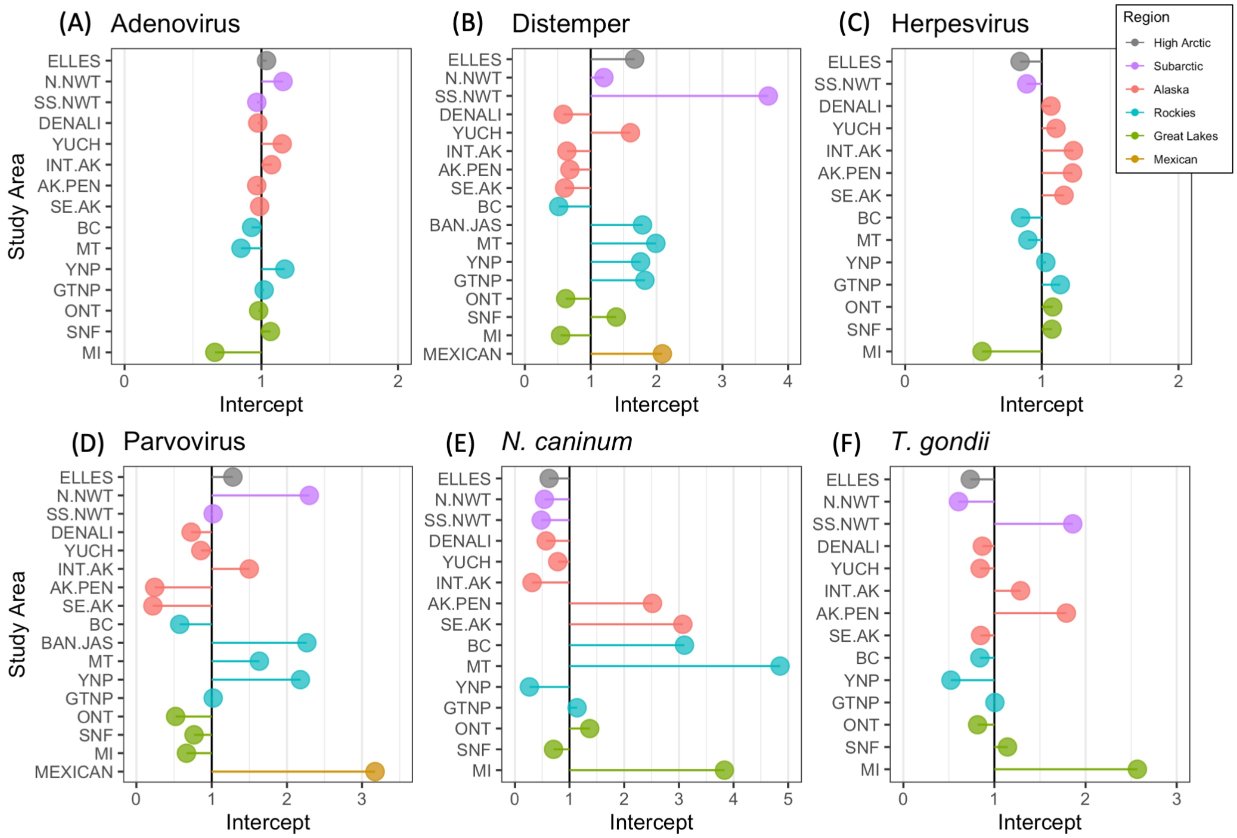

Figure S20. Plots of random intercept estimates for *complete models* predicting exposure to (A) adenovirus, (B) distemper, (C) herpesvirus, (D) parvovirus, (E) *N. caninum*, and (F) *T. gondii* for each study area. Intercept estimates are in terms of odds (i.e., exponentiated) such that values less than one indicate that wolves in that study area have lower odds of exposure than the average of all wolves sampled (i.e., grand mean intercept), and values greater than one have greater odds of exposure. Study areas are arranged from approximately north to south and grouped by region: High Arctic (gray), Subarctic (purple), Alaska (red), central Rocky Mountains (turquoise), Great Lakes (green), and Mexican (gold) (see Fig. 1 caption for study area abbreviations). Note that not all study areas were tested for each pathogen. We excluded *color* due to lack of data in some study areas.

Literature Cited

1. North American Land Change Monitoring System 30m, 2010-2015 (Landsat). *Commission of Environmental Cooperation* (2015). Available at: http://www.cec.org/north-american-land-change-monitoring-system/.
2. USGS EROS Archive - Digital Elevation - Global 30 Arc-Second Elevation (GTOPO30). *Earth Resources Observation and Science (EROS) Center* (1996).
3. Hesselbarth, M. H. K., Sciaini, M., With, K. A., Wiegand, K. & Nowosad, J. landscapemetrics: an open-source R tool to calculate landscape metrics. *Ecography.* **42**, 1648–1657 (2019).
4. Poole, K. G., Wakelyn, L. A. & Nicklen, P. N. Habitat selection by lynx in the Northwest Territories. *Can. J. Zool.* **74**, 845–850 (1996).
5. Nielsen, S. E., Boyce, M. S., Stenhouse, G. B. & Munro, R. H. M. Modeling grizzly bear habitats in the yellowhead ecosystem of Alberta: Taking autocorrelation seriously. *Ursus* **13**, 45–56 (2001).
6. Kittle, A. M. *et al.* Landscape-level Wolf space use is correlated with prey abundance, ease of mobility, and the distribution of prey habitat. *Ecosphere* **8**, (2017).
7. Morin, S. J., Bowman, J., Marrotte, R. R. & Fortin, M. J. Fine-scale habitat selection by sympatric Canada lynx and bobcat. *Ecol. Evol.* **10**, 9396–9409 (2020).
8. O'Neil, S. T., Vucetich, J. A., Beyer, D. E., Hoy, S. R. & Bump, J. K. Territoriality drives preemptive habitat selection in recovering wolves: Implications for carnivore conservation. *J. Anim. Ecol.* **89**, 1433–1447 (2020).
9. Arjo, W. M. & Pletscher, D. H. Coyote and wolf habitat use in northwestern Montana. *Northwest Sci.* **78**, 24–32 (2004).
10. Oakleaf, J. K. *et al.* Habitat selection by recolonizing wolves in the northern Rocky Mountains of the United States. *J. Wildl. Manage.* **70**, 554–563 (2006).
11. Hebblewhite, M. & Merrill, E. Modelling wildlife-human relationships for social species with mixed-effects resource selection models. *J. Appl. Ecol.* **45**, 834–844 (2008).
12. Roever, C. L., Boyce, M. S. & Stenhouse, G. B. Grizzly bears and forestry II: Grizzly bear habitat selection and conflicts with road placement. *For. Ecol. Manage.* **256**, 1262–1269 (2008).
13. Houle, M., Fortin, D., Dussault, C., Courtois, R. & Ouellet, J. P. Cumulative effects of forestry on habitat use by gray wolf (*Canis lupus*) in the boreal forest. *Landsc. Ecol.* **25**, 419–433 (2010).
14. Mayor, S. J., Schneider, D. C., Schaefer, J. A. & Mahoney, S. P. Habitat selection at multiple scales. *Ecoscience* **16**, 238–247 (2009).
15. Milakovic, B. *et al.* Habitat selection by a focal predator (*Canis lupus*) in a multiprey ecosystem of the northern Rockies. *J. Mammal.* **92**, 568–582 (2011).
16. Kittle, A. M. *et al.* Wolves adapt territory size, not pack size to local habitat quality. *J. Anim. Ecol.* **84**, 1177–1186 (2015).
17. NASA Socioeconomic Data and Applications Center. Gridded Population of the World (GPW), v4. *EarthData* (2015). Available at: https://sedac.ciesin.columbia.edu/data/set/gpw-v4-population-count-rev11/data-download.
18. Millán, J. *et al.* Patterns of exposure of Iberian wolves (*Canis lupus*) to canine viruses in human-dominated landscapes. *Ecohealth* **13**, 123–134 (2016).
